# Massive expansion of sex-specific SNPs, transposon-related elements, and neocentromere formation shape the young W-chromosome from the mosquitofish *Gambusia affinis*

**DOI:** 10.1186/s12915-023-01607-0

**Published:** 2023-05-15

**Authors:** Stefan Müller, Kang Du, Yann Guiguen, Maria Pichler, Shinichi Nakagawa, Matthias Stöck, Manfred Schartl, Dunja K. Lamatsch

**Affiliations:** 1 Institute of Human Genetics, Munich University Hospital, Ludwig Maximilians University, Munich, Germany; 2 Department of Chemistry and Biochemistry, The Xiphophorus Genetic Stock Center, Texas State University, San Marcos, TX USA; 3 INRAE, LPGP, Rennes, France; 4 Universität Innsbruck, Research Department for Limnology, Mondsee, Mondsee, Austria; 5 Evolution & Ecology Research Centre and School of Biological, Earth and Environmental Sciences, University of New South Wales, Sydney, Australia; 6 Leibniz-Institute for Freshwater Ecology and Inland Fisheries (IGB), Department of Ecophysiology and Aquaculture, Berlin, Germany; 7 Amphibian Research Center, Hiroshima University, Higashihiroshima, 739-8526 Japan; 8 Developmental Biochemistry, University of Würzburg, BiozentrumWürzburg, Germany

**Keywords:** Sex chromosome evolution, ZZ/ZW sex chromosomes, Transposable element, SNP accumulation, W-specific non-coding gonadal expression, Neocentromere

## Abstract

**Background:**

The Western mosquitofish, *Gambusia affinis*, is a model for sex chromosome organization and evolution of female heterogamety. We previously identified a *G. affinis* female-specific marker, orthologous to the aminomethyl transferase (*amt*) gene of the related platyfish (*Xiphophorus maculatus*). Here, we have analyzed the structure and differentiation of the *G. affinis* W-chromosome, using a cytogenomics and bioinformatics approach.

**Results:**

The long arm of the *G. affinis* W-chromosome (Wq) is highly enriched in dispersed repetitive sequences, but neither heterochromatic nor epigenetically silenced by hypermethylation. In line with this, Wq sequences are highly transcribed, including an active nucleolus organizing region (NOR). Female-specific SNPs and evolutionary young transposable elements were highly enriched and dispersed along the W-chromosome long arm, suggesting constrained recombination. Wq copy number expanded elements also include female-specific transcribed sequences from the *amt* locus with homology to TE. Collectively, the *G. affinis* W-chromosome is actively differentiating by sex-specific copy number expansion of transcribed TE-related elements, but not (yet) by extensive sequence divergence or gene decay.

**Conclusions:**

The *G. affinis* W-chromosome exhibits characteristic genomic properties of an evolutionary young sex chromosome. Strikingly, the observed sex-specific changes in the genomic landscape are confined to the W long arm, which is separated from the rest of the W-chromosome by a neocentromere acquired during sex chromosome evolution and may thus have become functionally insulated. In contrast, W short arm sequences were apparently shielded from repeat-driven differentiation, retained Z-chromosome like genomic features, and may have preserved pseudo-autosomal properties.

**Supplementary Information:**

The online version contains supplementary material available at 10.1186/s12915-023-01607-0.

## Background

Suppression of meiotic recombination between X- and Y- or Z- and W-chromosomes, at least around the master sex-determining gene, is one of the first events in the evolution of sex chromosomes. This is a precondition for sex chromosomal distinctiveness and initiates potential morphological differentiation [1]. The classical view is that Y- and W-chromosomes will continuously decrease in gene content and possibly even in size and may end up in highly conserved systems, as found in mammals and birds, as degenerated genomic chromosomal elements, which may be very small and sometimes heterochromatic [2–4].

In poikilothermic vertebrates like fishes [5], amphibians [6], and some reptiles [7], however, sex-determining mechanisms exhibit a greater diversity [8, 9]. In species with genetic sex determination, undifferentiated sex chromosomes predominate, and several evolutionary mechanisms explain this prevalence. These include turnovers, which alter the master sex determination gene, the sex chromosome, or the sex determination system (e.g., XY to WZ, [5, 10, 11]); jumping master genes that retain the main genetic triggers but translocate to other chromosomes [12, 13]; or occasional recombination (e.g. in sex-reversed females), which in some systems may prevent sex chromosome degeneration [14].

In teleost fish, even closely related species or different populations of the same species may have evolved contrasting ZW/ZZ or XX/XY sex chromosome systems [15–17], and only 10% of the karyotyped gonochoristic teleost species possess cytogenetically heteromorphic sex chromosomes [18]. In many cases, the Y- or W-chromosome of the heterogametic sex is larger than its X or Z counterpart. These size differences range from small to extreme cases, in which the W or Y is the largest element of the karyotype (for overview [1], Table 5 therein).

The Western mosquitofish, *Gambusia affinis*, [19], originating from North America, has 2*n* = 48 chromosomes and is female heterogametic (ZW/ZZ) [20–22]. With 45 Mbp, the W is the largest and also the only bi-armed, submetacentric element of the karyotype. Except for the smallest pair, all other chromosomes were identified as acrocentric, including the Z, which is the largest acrocentric chromosome with 28 Mbp in size [20–24]. The closely related *G*. *holbrooki*, with a divergence time of approximately 2–7 Ma ([22] and refs. therein) is morphologically almost indistinguishable from *G*. *affinis* but features an entirely acrocentric chromosome set with homomorphic sex chromosomes and an XX/XY sex determination system [21]. Of note, the sex chromosomes of *G. affinis* and *G. holbrooki* are not homologous, because the *G. affinis* WZ pair is homologous to platyfish LG 01, whereas the *G. holbrooki* XY corresponds to platyfish LG 16 [22].

Using a transcriptome-derived female-specific marker, we previously had developed a rapid PCR genotyping test for the identification of genetic sex in *G. affinis* [25, 26], with the ultimate goal to test the efficacy of introducing sex-reversed ZZ females as “Trojan individuals” into invasive populations of this species. It is hypothesized that this breeding strategy might result in the extinction of an entire population within a single or a few generations, because reproducing sex-reversed ZZ females with naturally occurring ZZ males would yield only ZZ males [27]. This possibility was investigated for conservation applications in New Zealand where this species was introduced for mosquito control and became an invasive species, as in many other parts of the world [28, 29]. Sequence analyses of this female-specific marker revealed high similarity with the 3′ UTR of the *aminomethyl transferase* (*amt*) gene of a related poecilid species, the platyfish (*Xiphophorus maculatus*).

The *amt* gene is a widely distributed and evolutionary conserved aminotransferase involved in glycine decarboxylation [30]. So far, *amt* has not been characterized as being involved in vertebrate sex determination or sexual development. We therefore studied the broader genomic context of the *amt* locus on the *G. affinis* W-chromosome using a comparative cytogenomics approach, to determine whether the amt locus resides within a fully W-linked region and thereby to elucidate its function. Both our FISH analyses and in silico data mining revealed that the long arm of the *G. affinis* W-chromosome (Wq) harboring the *amt* locus is highly enriched for evolutionary young copies of disperse repeats and has accumulated female-specific SNPs. Some repeats show partial homology to transposable elements (TE) that are also present in the *amt* gene region. At least one of these repetitive elements was found to be W-chromosome specific and expressed in ovary cDNA but not in the testis, just like *amt* itself. Moreover, these events were accompanied by a structural rearrangement of the W-chromosome, most presumably the formation of a neocentromere. Taken together, our results show that the *G. affinis* W-chromosome represents an exquisite example for a rapid sex chromosome differentiation by repeat accumulation.

## Results

### Large-scale genomic properties of the *G. affinis* W-chromosome

**Fig. 1:**
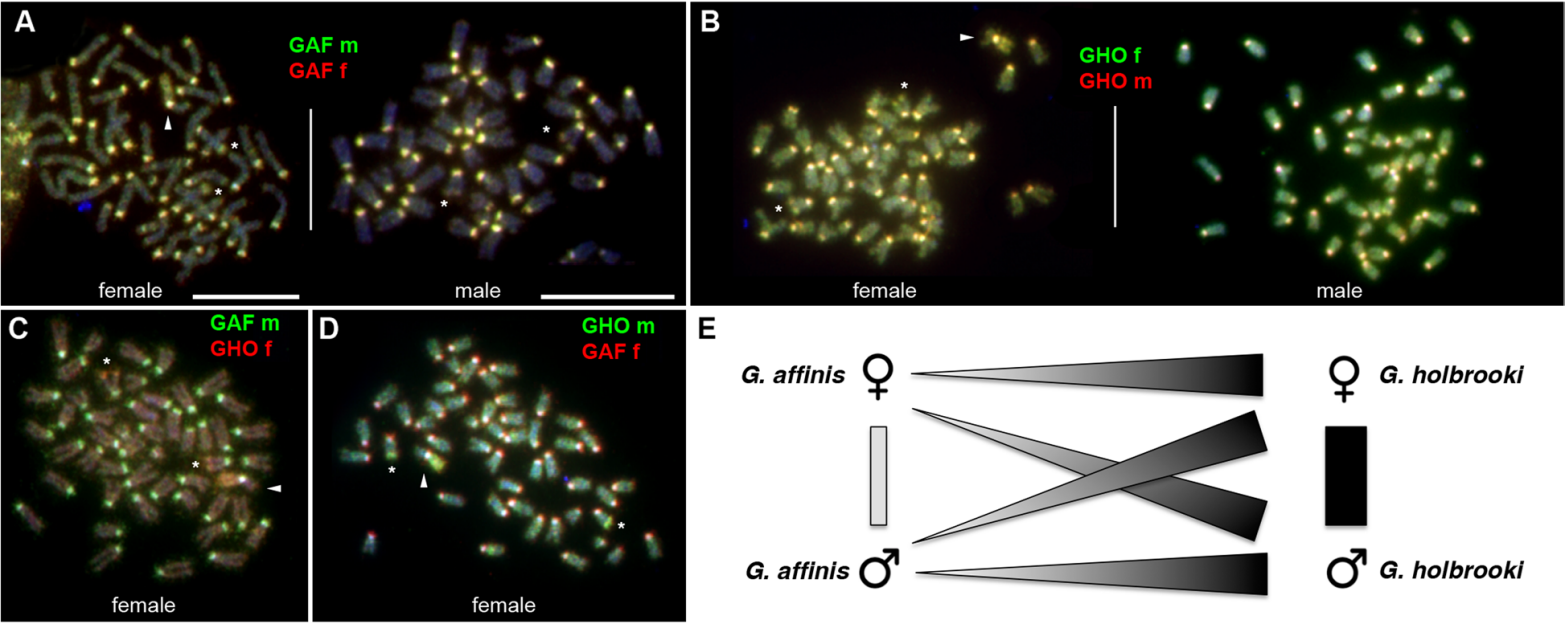
Comparative genomic hybridization (CGH) using genomic DNA from *Gambusia affinis* (GAF) and *G. holbrooki* (GHO) male (m) and female (f) individuals, hybridized to *G. affinis* metaphases. **A**–**D** Examples of *G. affinis* metaphase spreads after CGH using differentially labeled combinations of genomic DNA as indicated. Repetitive elements are enriched in the centromeric regions of all chromosomes. Arrowheads highlight the W-chromosome in female metaphase spreads, with the entire long arm Wq enriched in repeats present in males and females from both species. Asterisks mark a medium sized acrocentric pair in males and females showing a long-arm subtelomeric repeat cluster overrepresented in *G. holbrooki*. **E** Summary of genomic imbalances detected by CGH. Genome-wide copy number of repeat sequences is generally higher in *G. holbrooki* than in *G. affinis*, but no sex-specific copy number imbalances were observed in the two species (scale bars: 10 µm)

We performed comparative genomic hybridization (CGH) to recover sex- and species-specific genomic imbalances. Specifically, we delineated copy number differences between the genomes of male and female *G. affinis*, between male and female *G. holbrooki*, and between the genomes of the two species using the *G. affinis* chromosome set as reference. Technical details are outlined in the “Methods” section, and representative FISH images are shown in Fig. 1A–D and Fig. S1A, B. CGH using differentially labeled genomic DNA between *G. affinis* males and females revealed increased copy numbers of highly repetitive centromeric tandem repeats, shared by males and females in the centromeric regions of all chromosomes, compared to baseline intensities in single copy sequences, but no sex-specific differences. The long-arm subtelomeric regions of one presumably homologous pair of acrocentric chromosomes appeared less intense than the centromeric regions, but still showed clearly increased signal intensities in both sexes, likely harboring a major rDNA locus (Fig. 1, Fig. S1, see below). Notably, in female metaphases, the entire long arm of the W-chromosome (Wq) also showed increased FISH signal intensities from all genomic DNAs hybridized. These observations point to the accumulation of dispersed repetitive sequences of intermediate copy numbers in this region, although no sex-specific copy number differences could be detected on the W-chromosome at the resolution of CGH.

The same picture emerged when comparing homologous sequences from *G. holbrooki* XY males vs. XX female using *G. affinis* metaphases as a reference. Both male and female *G. holbrooki* genomes share repetitive sequences with *G. affinis*, enriched in centromeres, rDNA clusters, and Wq. However, no hallmarks of *G. holbrooki* XX vs. XY sex chromosome sequence copy number differences were found, confirming earlier studies pointing to homomorphic, undifferentiated XY in *G. holbrooki* (Fig. 1B). Figure 1E summarizes our observations on large-scale differences in repeat content when comparing male and female genomes from the two species of *Gambusia* by CGH.

Next, zooming in on the sex chromosomes at increased resolution and to delineate ZW homologous sequences in situ, we designed a *G. affinis* Z-chromosome specific oligopaint probe from the repeat-masked male *G. affinis* LG 01 sequence recently published. The male *G. affinis* LG 01 assembly was previously identified as the Z-chromosome because it aligned with the female *G. affinis* LG 01, the W-chromosome [24].

**Fig. 2:**
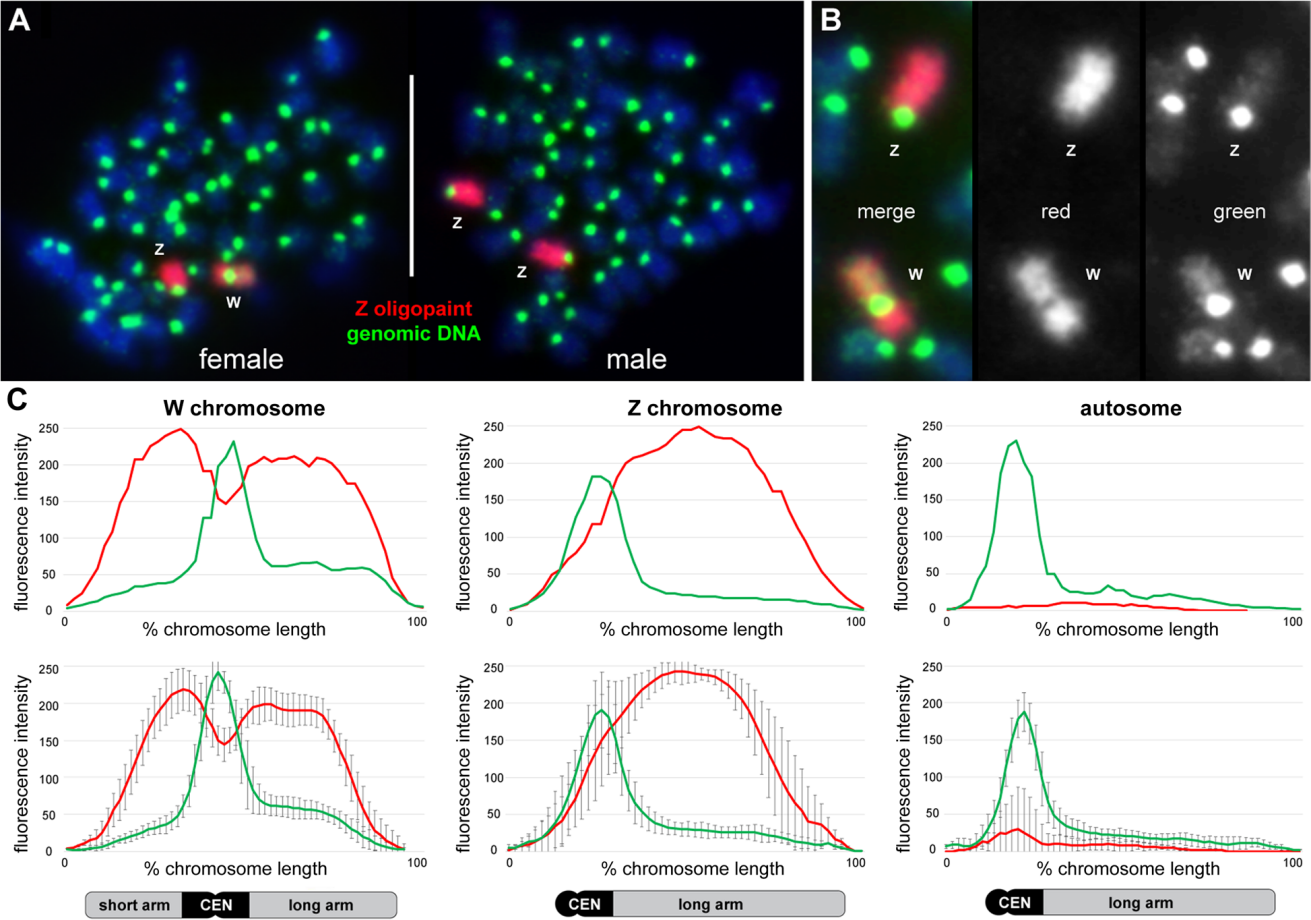
Quantitative chromosome painting in *G. affinis* using a *Z*-specific oligopaint probe. **A** Female (left) and male (right) *G. affinis* metaphases after co-hybridization of the GAF Z-oligopaint (red) with female genomic DNA (green). Note that the Z-oligopaint hybridizes to the Z- and the W-chromosome, indicating only very limited sequence divergence between Z and W. **B** enlarged partial metaphase—from left to right depicting the merged image, the Z-oligopaint, and the genomic DNA. **C** Quantitative fluorescence intensity profiling of the Z-oligopaint (red) and the genomic DNA (green) along the W-chromosome, the Z-chromosome, and an autosome. The top row illustrates the profile of each one individual chromosome from a single representative metaphase, the bottom row shows the mean profiles from ten metaphases. The Z-oligopaint shows higher fluorescence intensity on the Z-chromosome compared to the W. Moreover, oligopainting intensity on the W long arm was dimmed compared with the W short arm. Genomic DNA profiles showed high-intensity values in centromeric regions (CEN) of W, Z, and autosomes, as well as an increased hybridization intensity in euchromatic regions on Wq compared to Wp, Z, and autosomes

FISH using the Z-oligopaint probe in male metaphases showed the expected hybridization on a large pair of acrocentric chromosomes, decorating the entire chromosome arm and only excluding the centromere (Fig. 2A). Within the technical resolution limits of chromosome painting, no structural rearrangements were observed between the Z-chromosome and an autosome. Interestingly, when hybridized to female ZW metaphases, not only the Z-chromosome but also the W-chromosome appeared entirely fluorescently labeled from end to end (Fig. 2A, B). This demonstrates a very low overall sequence divergence between Z and W, in agreement with a measured sequence divergence of 1–2% (see below). Furthermore, this indicates large-scale conservation between Z and W from one chromosome end to the other, despite the facts that (i) the 45 Mbp W-chromosome is a much larger chromosome than the 28 Mbp Z and (ii) the submetacentric W is structurally distinctly different from the acrocentric Z. Again, no interchromosomal structural rearrangements were observed involving the Z- or the W-chromosome, effectively ruling out evolutionary gonosome-autosome translocations at the technical resolution of the assay and thus excluding the emergence of a neo-sex chromosome.

When quantitatively analyzing the paint hybridization pattern in comparison with genomic DNA by fluorescence intensity (FI) profiling (Fig. 2C), the Z-oligopaint showed higher fluorescence intensity on the Z than the W. Moreover, oligopainting intensity on Wq was dimmed compared with the W short arm. FI profiles from genomic DNA were the same as described for CGH (compare Fig. 1), with high intensity in centromeric regions of W, Z, and autosomes, as well as increased intensity in the euchromatic region on Wq compared to Wp, Z, and autosomes.

### Integrating sequence assembly and structure of the *G. affinis* W-chromosome

Before characterizing the sequence differentiation between the sex chromosomes in greater detail, we determined the structural orientation of the W-chromosome with respect to the published W-chromosome sequence assembly [24]. Unfortunately, we could not recover the centromeric region from the genome assembly of Shao et al. [24] because of obvious difficulties to assemble correctly this highly repetitive region of the W-chromosome. The region between 27.5 and 30.5 Mbp is represented in the genome assembly by a long stretch of “Ns” making it impossible to search, for instance, for the typical alpha-satellites. However, identification of the *amt* gene at a position in line with the FISH data unequivocally defines the long arm of the W (see below). In addition, we determined the position of the W centromere at 59.6% ± 2.2% total length by measuring the chromosome arm length ratio and then physically anchored the sequence assembly with cytogenetic reference points obtained by CGH and oligopainting. Given a total length of 45 Mbp, this would translate to a position of the centromere at 17.2–19.2 Mbp or at 25.8–27.8 Mbp, depending on the overall orientation of the assembled sequence. However, taking into account the CGH and oligopainting data, the hypothesis of a centromere positioning at 17.2–19.2 Mbp could be readily refuted. Firstly, by CGH, the entire long arm was proven to be enriched with disperse repeats, whereas no repeat-derived increase in CGH signal intensity was detectable on the short arm (Fig. 2C). Secondly, the oligopainting intensity was clearly dimmed on the entire q-arm compared to the p-arm (Fig. 2C). Assuming that the centromere would be located at 17.2–19.2 Mbp, it would be embedded in the repeat-rich stretch of sequences ranging from 0 to 27.5 Mbp of the W-assembly (see also below), and not at the border between high and low CGH and oligopainting signal intensities. These cytogenetic data are fully in agreement with the characteristic gap between 27.5 and 30.5 Mbp of the Hi-C data [24], likely corresponding to the position of the centromere, and related to the preferential intra-arm 3D-proximity, and the lack of close 3D-proximity between p- and q-arm chromatin, which is usually observed in bi-armed chromosomes ([31]; compare Fig. S2 in [24]).

### Repetitive landscape of the *G. affinis* genome

A series of classical and molecular cytogenetic assays, as well as immuno-fluorescence staining, provided further details of the genomic organization and the epigenetic and transcriptional properties of the *G. affinis* sex chromosomes.

We first probed the genome-wide distribution of ten common mono-, di-, and trinucleotide microsatellite repeat motives in five dual-color FISH experiments. In essence, the *G. affinis* genome, and in particular the W-chromosome, showed no clusters of (C)_30_, (A)_30_, (GA)_15_, (CA)_15_, (CAC)_10_, (TA)_15_, (CGG)_10_, (GAG)_10_, (GAC)_10_, or (CAT)_10_ repeat arrays detectable by FISH (Fig. S2A-C). Only some acrocentric chromosomes showed slight proximal enrichment of poly-T repeats (Fig. S2A), and Wq distally harbors a stretch enriched for CGG-repeats (Fig. S2C).

**Fig. 3:**
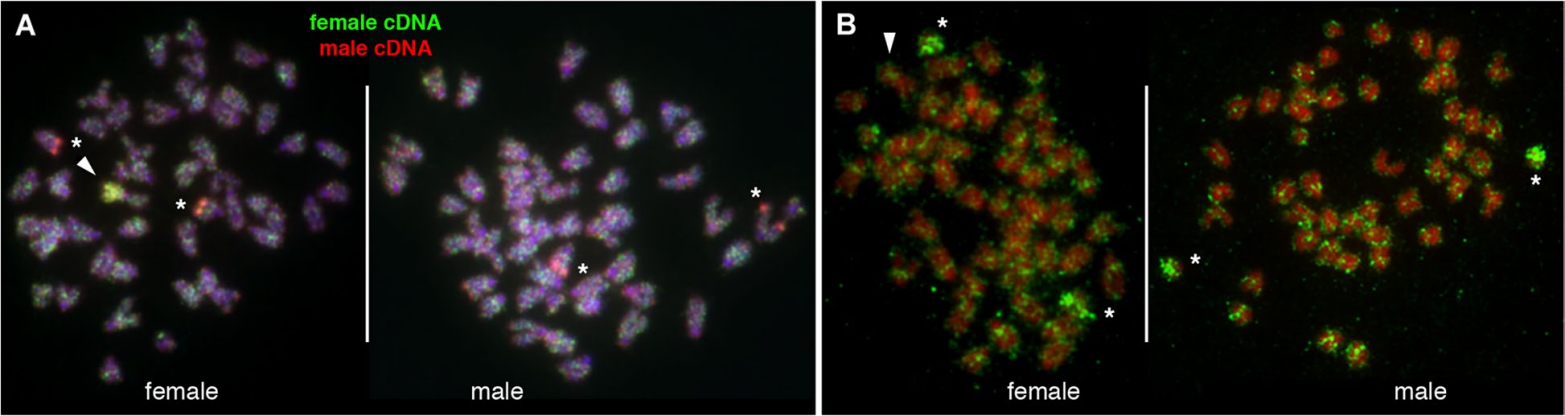
**A** Comparative expressed sequence hybridization (CESH) of differentially labeled testis and ovary cDNA libraries demonstrates expression of repetitive elements from both sexes enriched along the entire Wq (arrowhead). In addition, a medium-sized acrocentric pair in females (left) and males (right) showed a male-expressed repeat cluster in the long-arm subtelomeric region (asterisks). **B** Immuno-fluorescence staining of hypermethylated DNA using an anti-5mC antibody showed no enrichment of DNA methylation of the W-chromosome (arrowhead) indicating that the Wq region does not comprise transcriptionally silenced chromatin. Intriguingly, in both female (left) and in male (right) metaphases, a presumably homologous chromosome pair of autosomes was entirely hypermethylated in all analyzed mitoses (asterisks)

Combined staining of *G. affinis* chromosomes using DAPI (AT-rich chromatin) and 7-AAD (GC-rich chromatin) showed a trend towards CG-rich pericentromeric and proximal chromosome regions and more AT-rich chromatin at distal q-arms (Fig. S2D, Fig. S3). Sequential DAPI/7-AAD karyotyping followed by FISH using the Z-oligopaint and gDNA confirmed that the Z-chromosome is the largest acrocentric and that the W-chromosome is approximately 20% larger than the Z (Fig. S3). The W-chromosome short arm showed higher AT-content compared to the more intensely 7-AAD stained W long (q-) arm, indicating homology between the W short arm and the distal segment of the Z (Fig. S3D). The Wq arm subtelomere is very GC-rich, in line with the presence of an rDNA cluster, whereas the putative homologous region at the Z subtelomere is AT-rich, possibly lacking a NOR (Fig. S3, see also below). In addition, the long-arm subtelomere of a medium-sized pair of acrocentric autosomes was found to be extremely CG-rich, likely corresponding to another rDNA cluster (Fig. S3C, compare with Figs. 1 and 3), and the smallest pair was confirmed as being submetacentric.

C-banding in *G. affinis* revealed constitutively heterochromatic regions restricted to all centromeric regions, including the W-chromosome (Fig. S2E). Alike all other chromosomes, the entire Wq was C-banding negative.

### Epigenetic and transcriptional features of the *G. affinis* W-chromosome

Ag-NOR staining demonstrated the subterminal region of Wq to harbor an active NOR (Fig. S2F and G). Active NORs were found on three to six chromosomes per metaphase (*n* = 19 metaphases analyzed; mean female 3.9, mean male 4.8), including the short p-arms of 2–4 acrocentrics, q-arm subtelomeres on 1–2 acrocentrics, and the Wq subtelomeric region (Wqter). By FISH using PCR-products amplified from a mixture of *G. affinis* male and female genomic DNA, and universal metazoan 28S rDNA primer pair 8F/11RC [32], Wqter, the p-arm satellites of up to four acrocentrics, and q-arm subtelomeric regions on up to two acrocentrics showed rDNA FISH signals (*n* = 20 metaphases analyzed; mean female 3.2, mean male 3.8). However, 28S rDNA FISH signals were difficult to discern owing to co-hybridization with all centromeric regions (Fig. S2H). Replacement with PCR-primers GAF28S-L1/R1 designed from *G. affinis* rDNA sequence did not improve specificity (Fig. S2I).

CESH was employed to obtain a chromosome-centered view about differentially transcribed genomic regions in *G. affinis* gonadal tissues. CESH is a derivative of the CGH technique and allows for the transcriptome-wide detection and chromosomal assignment of large-scale expression differences [33]. Here, we combined differentially labeled, reverse-transcribed total RNA from the testis and ovary tissue from our previous study [26] in the CESH assay. CESH revealed that the entire Wq comprises DNA sequences highly expressed in both, ovary and testis (Fig. 3A). Another highly expressed region was observed in the q-arm subtelomeric regions of one presumably homologous pair of autosomes, apparently overexpressed in the testis compared to the ovary. The latter CESH signal likely corresponds to rDNA transcripts from a major autosomal rDNA locus, also identified by CGH and Ag-NOR staining (compare Fig. 1, Fig. S2F and G). No other chromosomal regions were found to be transcriptionally highly upregulated or differentially expressed in male or female mosquito fishes. Of note, hybridization to centromeric DNA was absent.

We then visualized 5-methylcytosine (5mC) transcriptionally repressive marks in *G. affinis* mitotic cells by immune-fluorescent staining employing an anti-5-methylcytosine (anti-5mC) antibody. The W-chromosome showed no anti-5mC fluorescent staining above background levels (Fig. 3B) in any metaphase. Strikingly, a presumed pair of autosomal homologs, as determined by similar size and DAPI staining pattern, was found at least in large parts, and possibly in its entirety, to be labeled by anti-5mC immune fluorescence, both in male and in female metaphases (Fig. 3B).

### Sequence divergence and gene decay, SNP density, and TE expansion

**Fig. 4:**
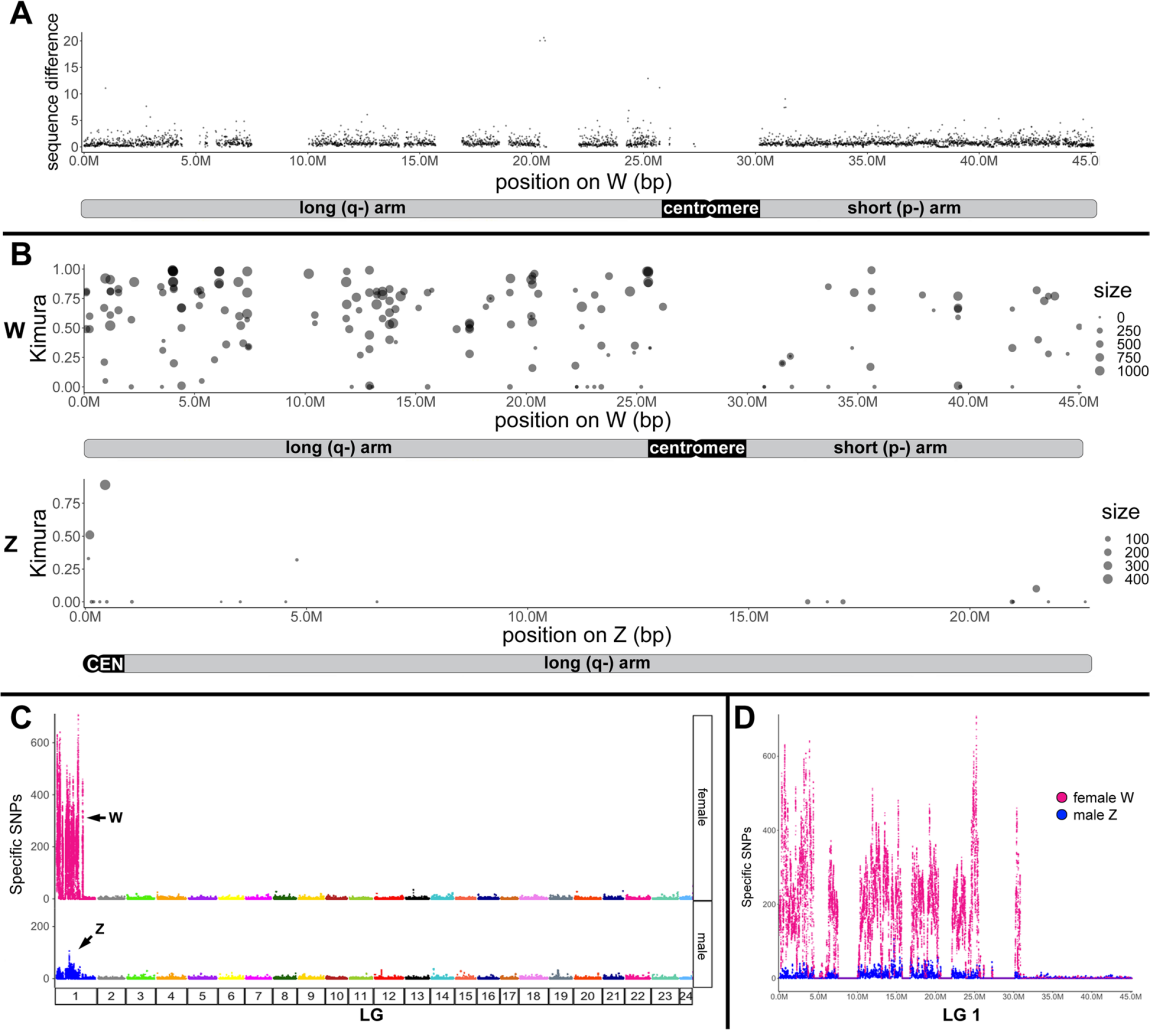
Sequence differences, distribution of repeat elements, and abundance of sex-specific SNPs on the W- and Z-chromosomes of *Gambusia affinis* and integration with cytogenomic data. The gaps in the W sequences correspond to stretches of N’s in the published genome assembly [24]. **A** Sequence difference between W and Z, using W as a reference. **B** Location of young repeats (with kimura < 1) on W (top panel) and on Z (bottom panel) shows that the W-chromosome, and specifically the long arm Wq, is highly enriched in young repeats compared to Z. **C**, **D** The molecular differentiation of the sex chromosomes of *G. affinis* demonstrates a massive increase in sex-specific single-nucleotide polymorphisms (SNPs) on the W-chromosome compared to autosomes and the Z-chromosome. **C** Plots showing the number of female- and male-specific SNPs along the 24 chromosomes. **D** Quantitative analysis of female-specific (magenta) and male-specific (blue) SNPs along female and male LG 01, the W- and Z-chromosome, respectively, using the W-chromosome as a reference

Next, we calculated the sequence difference as the percentage of mutations in 10 kb sliding windows (Fig. 4A). This revealed a sequence difference to the corresponding region of the Z for the W long arm at 0.93% (1st quarter to 3rd quarter: 0.43–1.13%; median: 0.66%) and for the W short arm at 0.86% (1st quarter to 3rd quarter: 0.46–1.04%; median: 0.70%). Thus, no region with elevated sequence difference was apparent.

We also calculated the extent of gene decay between the long (0–27.5 Mbp) and short arm (27.5–45 Mbp) of the W-chromosome. From a collection of 455,817 protein sequences retrieved from the vertebrate database of Swiss-Prot, 1.284 protein-coding genes were identified on the * G. affinis* W with an alignment coverage > 35% and percentage of identity > 50%. On the Z-chromosome, 1046 potential protein-coding genes (PCGs) were retrieved (quality control: alignment coverage > 35% and percentage of identity > 50%). We then implemented a gene cluster analysis based on sequence similarity (*H*-score > 90) and found 21 PCGs of Z produced in a total of 44 copies on W (Table S1). Thirty-five of these are located on the long arm and 9 on the short arm. We then lifted these potential PCGs onto W and only two failed to be lifted. When we blasted the two failed PCGs on NCBI, the best hits were “hypothetical protein CCH79_00020665, partial [Gambusia affinis]” and “hypothetical protein CCH79_00018692, partial [Gambusia affinis]” respectively.

Out of the 690 genes on the W long arm, 144 (20.9%) have a frameshift or premature stop codon and thus represent pseudogenes. On the short arm, only 97 out of 594 genes (16.3%) represent pseudogenes. By the same method, we identified 151 (14.4%) pseudogenes out of the 1062 potential PCGs for the Z and estimated a 12.5% average pseudogene content for autosomes. This 8.4% increase of pseudogenes on the W long arm compared to autosomes is non-negligible. However, it represents a rather moderate level of gene decay compared to the more than 30 million years old W-chromosome of the tongue sole, which has already lost two-thirds of the approximately 900 genes preserved on its Z counterpart, and which is enriched for pseudogenes by 17.2% compared to autosomes (19.7% on W, 3.5% on Z, and 2.5% on autosomes) [34].

A completely different picture emerged when repetitive elements were considered. A previous study has reported a recent burst of TEs on the W-chromosome of *G. affinis* [24]. To characterize the spatial distribution of TEs on the W, we retrieved the fraction of TEs which are assigned a young age by a Kimura value < 1 and located them on W- and Z-chromosome (Fig. 4B). Only very few young TEs are present on the Z, in contrast to a very high density on the W. Moreover, the distribution of repeats across the W-chromosome is not uniform. The proximal part (0–25 Mbp) of the W, corresponding to the long arm where the *amt* gene is centrally located, has a much higher density compared with the distal segment (30–45 Mbp) representing the W short arm. Out of 13,319 TEs on the long arm, 149 (1%) were identified as young TEs with Kimura < 1, which is almost three-fold of that (36/9115 = 0.39%) on the short arm.

For another sensitive measure to determine sequence differences between the W- and Z-chromosomes, we mapped the sequences from pools of 30 males and 30 females [22] to the W- and Z-chromosomes from the [24] assembly. On autosomes (LG 02-LG 24), the sex-specific SNP density in males and in females was altogether very low (Fig. 4C). Both sex chromosomes showed alleviated SNP levels, but the Wq stood out by an increase in factor of 10–20 compared to Z (Fig. 4C, D). Strikingly, when comparing sex-specific SNP levels between Z and W, the long arm of the W-chromosome (region 0–25 Mbp) is highly enriched for female-specific SNPs, overlapping with the region where the recent expansion of TEs was mapped, plus a short segment at 30 Mb corresponding with the W short arm pericentromeric region (Fig. 4D).

### Molecular cytogenetic analysis of the *G. affinisamt* locus

**Fig. 5:**
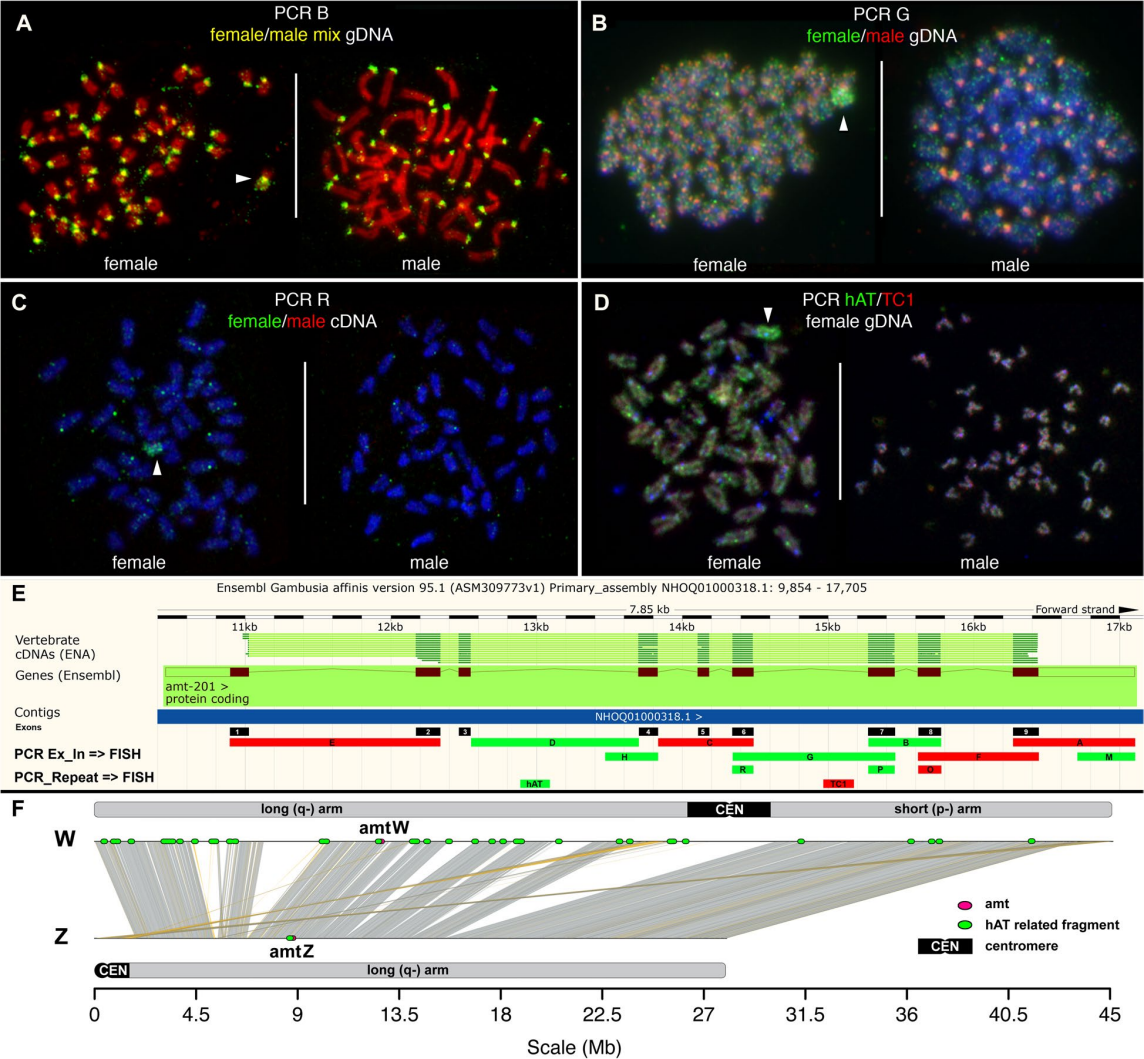
**A**–**D** FISH mapping of fluorescent PCR products from exonic and intronic regions of the *amt* locus to female and male *G. affinis* metaphases. **E** Summary of the results together with the Ensembl map detailing exon/intron structure of the gene. Green bars indicate PCR probes B, D, G, H, M, R, P, and hAT, yielding FISH signals on *Gambusia* Wq. Red bars correspond to PCR probes A, C, E, F, O, and TC1 without FISH signals on Wq. **F** Synteny map of W and Z with the location of the *amt* gene on Wq (amtW) and on Z (amtZ), each shown by a red dot. Green dots indicate TE-related repeats with partial homology to hAT, Helitron, and MuDR that were returned from a BLAST search, using the hAT-related sequence from the *amt* locus as input, and that were also identified by FISH using PCR products D and hAT. Both sequence analysis and FISH showed high enrichment of these repeats on the long arm of W, and absence on Z

Since a previously identified female-specific *amt* homolog [26] is located close to the putative *G. affinis* SD locus (< 0.87 cM) on the W-chromosome, or may be even part of it, we further analyzed the genomic region of the *amt* locus by mapping exonic and intronic PCR probes from the region by metaphase FISH. Images from representative FISH experiments are shown in Fig. 5A–D and Fig. S3, and a summary of the *amt* gene map including all *amt*-PCR FISH results is given in Fig. 5E.

In the first set of experiments, *amt* gene segments A (primers 9F/Gaf88R), B (7F/8R), C (4F/6R), D (Int3F/Int3R), E (1F/2R), F (8F/9R), G (6F/7R), H (Int3F/4R), and M (Gaf88F/Gaf88R) were PCR amplified from a mixture of gDNA from five *G. affinis* individuals (three females from mix GAFf and two males from mix GAFm) and hybridized in situ to *G. affinis* metaphases. These primer sequences were previously described [26]. Every PCR product hybridized to all centromeres, both in males and in females. Importantly, PCR products B, D, G, H, and M additionally decorated the entire long arm of the W-chromosome. Representative FISH examples are shown in Fig. 5A and Fig. S4A, and the summary is given in Fig. 5E.

We then examined whether the *amt* genomic structure and chromosomal localization would follow a sex-specific pattern. For addressing this issue, we PCR amplified several *amt* regions separately from female gDNA mix GAFf and male mix GAFm, respectively. We repeated the PCR from interval G (primers 6F/7R) and also individually PCR amplified exons 6, 7, and 8, representing intervals O (8F/8R), P (7F/7R), and R (6R/6F). The respective male and female PCR products were differentially labeled and combined in FISH. The PCR product obtained from interval O (exon 8) showed no FISH signal from males or females, whereas PCR products G, P, and R from exons 6–7 showed a clear female-specific hybridization pattern decorating the entire Wq. After FISH, PCR-products G, P, and R from male templates only labeled centromeric regions, while amplification from female gDNA was barely detectable at centromeres, but specific to the entire Wq, indicating the presence of female enriched disperse repeats. Examples for FISH are illustrated in Fig. 5B and Fig. S4B.

To determine whether these Wq-enriched dispersed repetitive elements would be expressed, we repeated PCR reactions B, G, O, P, and R but used as a template the testis- or ovary-specific cDNA library from [26]. After differential labeling and FISH, we could show that only PCR products from ovary cDNA but not from testis cDNA yielded FISH signals on Wq (Fig. 5C). As in the CESH assay, cross-hybridization to centromeric DNA was not observed (Fig. 3A).

In summary, the long arm of the *G. affinis* W-chromosome harbors multiple copies of dispersed repetitive elements also found in certain segments of the *amt* locus, which are enriched in and are possibly specific to female and ovary-expressed DNA. These *amt -*segments include exons 4, 6, 7, and 8, introns 3, 6, and 7, and the 5′-UTR, corresponding to PCR products B, D, G, H, M, P, and R (Fig. 5E) and likely represent a subset of transcribed sequences also detected by CESH (Fig. 3A).

To identify these Wq-enriched disperse repeats, we searched the published *G. affinis* 6498 bp *amt* gene sequence from [26] for simple repeats, TEs, long non-coding RNAs, and micro-RNAs in public databases. We identified three micro-RNAs, six sequences with partial homology to human (5) or mouse (1) long non-coding RNAs, eight sequences with partial homology to TEs, and three simple repeats, totaling 1680 bp (24%) (Table S2). The following repeats corresponded to sequences included in PCR-products B, D, G, H, M, P, and R showing dispersed repetitive FISH localization along Wq (Fig. 5, Fig. S4, summarized in Fig. 5E): a simple repeat from intron 6, seven TEs (hAT-N145_DR, DNA/hAT-Charlie and Gypsy-9_OD-I from intron 3, Gypsy-14_GA-I, TC1_PP and Helitron-like-4a_Hmel from intron 6, and DNA-8-3_HM from intron 9), micro-RNA miR-19 from intron 3, and long non-coding human lncRNA NONHSAT070712 homolog as part of a lncRNA cluster covering the region between intron 3 and intron 5, and human NONHSAT097211 homolog from exon 7.

**Fig. 6:**
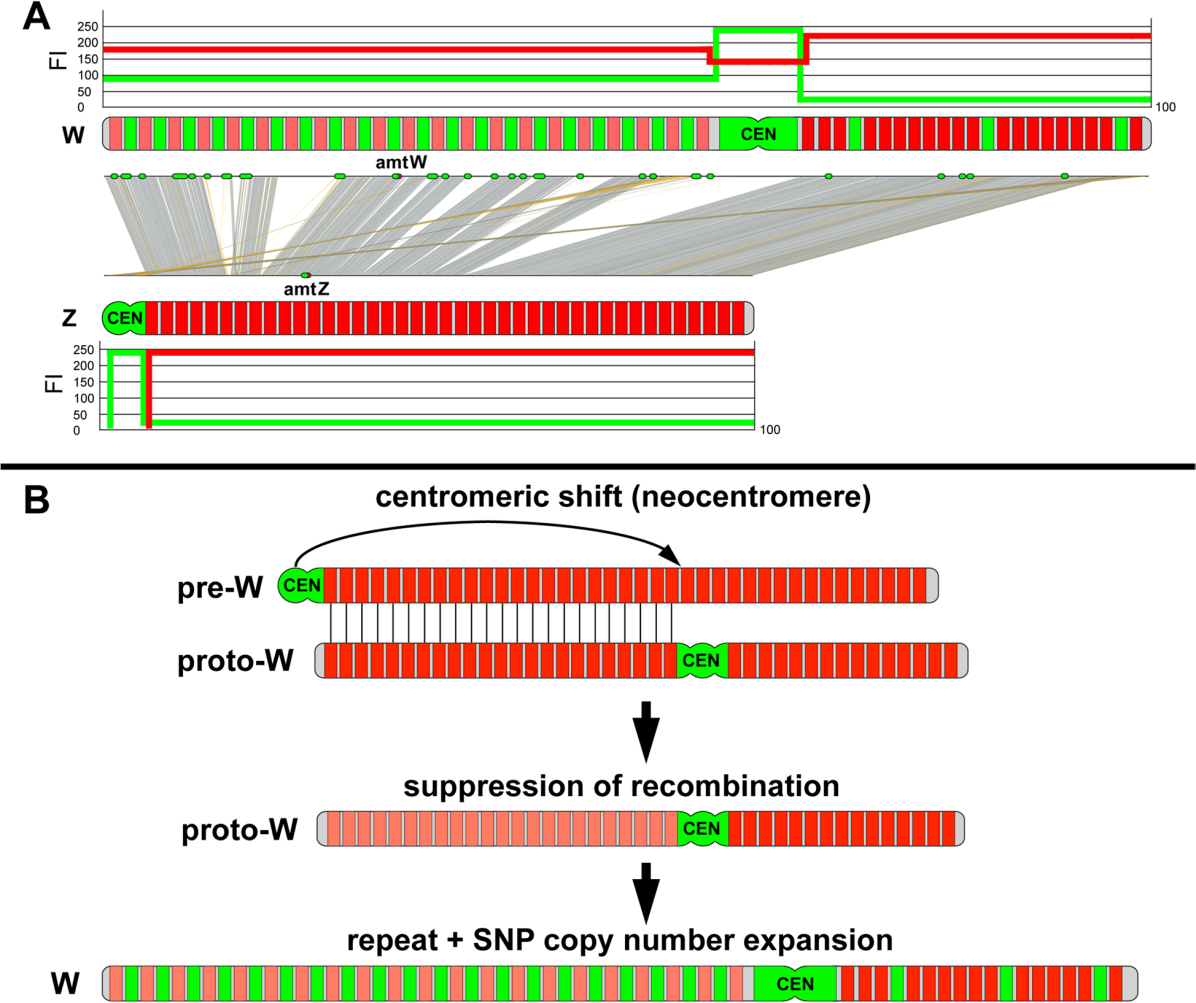
Integration of cytogenetic and sequence data, together with a reconstruction of the W-chromosome differentiation in *G. affinis*. **A** The acrocentric Z-chromosome shows low abundance of sex-specific SNPs and repeat elements including TEs (green bars), except for the centromere (CEN). Z is comparable to the autosomes, with low green fluorescence intensity (FI) values after in situ hybridization with green fluorescent genomic DNA, whereas oligopainting using a pool of evenly spaced red fluorescent oligonucleotides designed from the male LG 01 assembly (red bars) yielded high FI values on Z, except for CEN. The short arm of the W-chromosome, with high sequence homology to Z and with similar repeat density and red fluorescent paint oligo spacing, showed FI profiles comparable to Z. In contrast, on Wq, the massive accumulation of female-specific SNPs and of repeat elements corresponded with enhanced FI values from green fluorescent genomic DNA and with diminished FI values from red fluorescent Z-oligopaint probe because of higher spacing between adjacent oligos. **B** Stepwise reconstruction of the inferred most parsimonious pathway of evolutionary W-chromosome differentiation, starting from a pre-W resembling an acrocentric autosome: in the first step, the pre-W becomes structurally rearranged by a centromeric shift concomitant with neocentromere formation. Consequently, recombination is suppressed in the heterozygous Wq segment of the structurally derived proto-W (represented by pale red bars), followed by the accumulation of repeats including TE, and of female-specific SNPs

For a subsequent FISH validation, we selected two candidates, hAT-N145_DR and TC1_PP, for which it was possible to design PCR primers yielding amplification products of the appropriate size to be employed as probes. FISH using the TC1_PP PCR-amplification probe yielded no specific signal, indicating that product size and/or target copy number in the *G. affinis* genome is too small to be detected by FISH (Fig. 5D, E). In contrast, the hAT-N145_DR probe hybridized to the entire Wq-arm when PCR amplified from female *G. affinis* gDNA. We sequenced the FISH-positive hAT N145_DR PCR product, to elucidate whether the hAT-related fragment is part of a larger cassette of compound sequences distributed in multiple copies along the *G. affinis* Wq. We obtained 292 bp of sequence including 90 bp homologous to hAT-N145_DR. Blast analyses using this 90 bp fragment as a query returned a single hit on the Z-chromosome, which is at the homologous position in intron 3 of *amt*. On the W-chromosome, 265 hits outside the *amt* gene were found, all matching to longer hAT-N145_DR-related sequences. Strikingly, this hAT-N145_DR related fragment maps immediately adjacent to another approximately 400 bp stretch of repetitive DNA from intron 3, which is evolutionary conserved in multiple copies in several other fishes, including *Poecilia* and *Xiphophorus*, and which harbors a 350 bp fragment with partial homology to a hAT-related element from the Amazon molly, *Poecilia formosa* (Amazon_molly_rnd-4_family-1248#DNA/hAT-Charlie; http://www.fishtedb.org; Table S2). Consistent with the FISH results (Fig. 5D, E) and the mapping of young TEs, 97.7% were located in the region 0–25 Mbp corresponding to the Wq arm (Figs. 4B, 5F, and 6A).

Despite the *amt* locus containing repeat sequences, the gene itself is a single copy gene on the W. We used Exonerate (https://www.ebi.ac.uk/about/vertebrate-genomics/software/exonerate) to align the protein sequence of *amt* from Southern platyfish to the published female *Gambusia* assembly ([24]; chromosome W kept while Z removed). Only a single complete *amt* gene was found on the W, and no additional hits were obtained that could result from fragments of the gene on the W. We did the same alignment on chromosome Z, and again, only one complete *amt* copy was found (Fig. 5F).

## Discussion

Using a broad spectrum of cytogenetic, genomics and bioinformatic tools our analyses provided novel insights into the large-scale genomic structure of the evolutionary young *G. affinis* W-chromosome compared to the closely related male heterogametic *G. holbrooki*.

### The *G. affinis* W-chromosome is an early-stage sex chromosome

The identity and structure of sex chromosomes can be delineated by CGH, provided that sex chromosomes have been sufficiently differentiated and that copy number imbalances are involved in this process. The simple fact that the *G. affinis* W-chromosome is the largest chromosome in the complement—at least 20% larger than any of the acrocentric Z candidates—led us to speculate that the CGH approach may be feasible, because the estimated size of the 2:1 ZZ:ZW Z copy number difference would be well above the 10-Mbp technical resolution of CGH [35]. Although a 17-Mbp size difference between female LG 01 (W; 45 Mbp) and male LG 01 (Z; 28 Mbp) was recently determined at the sequence level [24], to our surprise, CGH mapping of genomic imbalances revealed no sex-specific copy number differences (Fig. 1, Fig. S1), suggesting that the molecular differentiation between *G. affinis* Z and W has not yet progressed to a stage where sufficient sex-specific copy number changes were accumulated to be detected by CGH. However, we observed an enrichment in repetitive sequences on the long arm of the *G. affinis* W-chromosome, also present elsewhere in the genome of male and female *G. affinis*, as well as in *G. holbrooki* (Fig. 1). The latter findings led us to conclude that, irrespective of sex, the *G. holbrooki* genome has a higher overall repeat load than *G. affinis*, including those repeats enriched on the long arm of the *G. affinis* W. Our CGH assays also confirmed the absence of cytogenetically differentiated X- and Y-chromosomes in *G. holbrooki* [21].

Oligopainting using a *G. affinis* Z-specific probe fully confirmed and extended these findings, hybridizing both Z and W along their entire length and only excluding the centromeres, thus—at the resolution of chromosome painting—demonstrating high sequence similarity from end to end (Fig. 2). Sequence alignment of male LG 01 and female LG 01, corresponding to Z- and W-chromosomes, respectively, showed the same pattern ([24] and this study, Fig. 5H). However, the sequence data alone provided no information about the obvious large-scale differences in genome structure between the submetacentric W and the significantly smaller acrocentric Z, nor about large gaps in the W sequence, resulting in an apparent 17-Mbp size difference between Z and W (Fig. 5F). Importantly, the diminished Z-oligopainting FISH intensity on Wq compared to Wp and Z complements the CGH data and suggests an enrichment of disperse repetitive elements on Wq not present in the pool of fluorescent oligonucleotides from the Z paint probe. In contrast, the observed very low W/Z sequence divergence of 1–2% without pronounced regional differences (Fig. 4A) is not sufficient to explain these findings. Unfortunately, oligopainting is not suitable to detect submicroscopic gonosome-autosome translocations below the technical limit of resolution, and the painting of entire chromosomes also misses intrachromosomal rearrangements such as inversions. Whole-genome sequencing, however, already indicated the co-linearity of W and Z and the absence of inversions or insertions on the W-chromosome.

### The W-chromosome is transcriptionally active and not heterochromatic

Despite the enrichment of repeats, the W long arm is C-banding negative (Fig. S2E), confirming the absence of constitutive heterochromatin [1, 22]. On the contrary, the presence of an active NOR and, more importantly, our CESH results (Fig. S2F, Fig. 3A) indicate that large segments of Wq are enriched in expressed sequences. This is in stark contrast to the more advanced stage of sex chromosome differentiation observed in *Leporinus*, an iconic model for sex chromosome evolution, where the Z is almost entirely heterochromatic and thus transcriptionally inert [36].

Expression of TEs in the gonads is usually high and reflects the characteristics of mobile DNA elements [37], and indeed, we and others found highly abundant evolutionary young TEs ([24], this study), which we could assign to the long arm of the W (Figs. 4, 5, and 6). Future studies on the identity and function of these W-transcripts should address if this high level of gonadal expression is connected to TE mobility. On the one hand, TE activity could become injurious by new deleterious insertions into the genome, but on the other hand, it may contribute to evolutionary advantageous regulatory effects [37]. These observations in the *G. affinis* W appear analogous to a recent finding for the Y-chromosome of the spotted knifejaw fish, exhibiting transcriptionally highly active testis-specific expression [38]. It was even hypothesized that this sex-biased expression assigns a “sex-beneficial” role for affected genes and may thus be a potent driver of sex chromosome evolution.

Conversely, and in line with high levels of expression from Wq, the anti-5mC immune-fluorescence assay showed Wq to be devoid of 5-methylcytosine clusters. DNA-methylation produces 5mC marks by methylation of cytosine residues, leading to repressed transcription, indicating that the *G. affinis* W long arm lacks epigenetically silenced chromatin. Surprisingly, in males and females, two acrocentric chromosomes of similar size and DAPI staining pattern showed abundant anti-5mC labeling (Fig. 3B). These autosomal homologs appear completely hypermethylated and therefore transcriptionally silenced. While functional implications remain speculative at this stage, future validation at high resolution, e.g., by methylome sequencing, is recommended in *G. affinis* and its relatives *G. quadruncus* and *G. speciosa* [39].

### Female enriched repeats from the *G. affinisamt* locus

The published *amt* sequence (Genbank KP113677) [26] includes simple repeats, TEs, long non-coding RNAs, and micro-RNAs (Table S2). The dispersed FISH localization of *amt* PCR products along Wq indicated an enrichment of certain repeats on the W-chromosome (Fig. 5A, B, Fig. S4 and summary in Fig. 5E). Furthermore, some of these repeats were found to be highly predominant in the female genome and perhaps even absent from the male (Fig. 5B, Fig. S4B). Moreover, RT-PCR FISH from the ovary and not from the testis cDNA hybridized to Wq, showing that the respective sequences are sex differentially transcribed (Figs. 3A and 5C). The ovary-specific expression in *G. affinis* resembles the testis-specific expression pattern of genes from the knifejaw fish neo-Y [40]. This remarkable preferential expression may be interpreted as a sex-beneficial function for some W-linked sequences. For a hAT-N145_DR TE-related repeat fragment from intron 3, a highly female-specific enrichment with accumulation on Wq was confirmed at the sequence level (Fig. 5F). hAT N145_DR from the hAT superfamily of TEs, named after “hobo” elements from *Drosophila*, “Ac” from maize, and Tam3 from snapdragon [41, 42], has not been implicated in sex chromosome evolution to date. However, our findings point to female-specific accumulation and expression of these disperse repetitive elements, hence contributing to W-chromosome structural and functional differentiation in *G. affinis*.

Concerning a possible role of *amt* in sexual determination or development, the data presented here allow the following conclusions: Although repeats present at the *amt* locus show dramatic sex-specific copy number expansion on Wq and also expression in the ovary but not in the testis (Figs. 3, 4, and 5), the gene itself may be non-essential to sex chromosome evolution or sex determination. As we showed, mostly intronic parts of the gene show this Wq disperse repetitive, multicopy, and sex-specific pattern. Parsimoniously, *amt* appears as an “innocent bystander” that descended into the maelstrom of repeat amplification and dispersal, rather than a driver of these dynamic evolutionary changes. This sex-specific locus merely mirrors the genomic landscape of the W long arm.

### Accumulation of sex-specific genomic features shaped the long arm of the W-chromosome

A very recent accumulation of transposable elements on the W was attributed to the size difference, with the Helitron superfamily as a major player in the sequence expansion on the W-chromosome. The hAT superfamily was also found to contribute significantly to the repeat content of the *G. affinis* genome, with over 174,000 copies and almost 30 Mbp (4.3%) of genomic sequence in females [24].

Our own analysis of the published reference sequence confirmed that the largest linkage group from the female genome (female LG 01, 45 Mbp) corresponds to the W, and the 28 Mbp LG 01 from the male genome to the Z. Our data analysis further indicates that the long arm of the W-chromosome (Wq) has been dramatically enriched in these young transposable elements. The copy number expansion of hAT-related elements may provide a case in point that this element is representative of the genomic landscape on Wq, suggesting a causal relationship between copy expansion of repetitive elements and non-coding RNAs from within the *amt* sequence and their multicopy distribution specifically along Wq.

These TE-related repeats were also highly enriched in the female genome, in particular on Wq (Figs. 4 and 5). Taken together, the massive copy number expansion of these TEs has contributed to the expansion of Wq. In contrast, the W short arm is only moderately populated by repeats, but still more densely than the Z or the autosomes. Strikingly, the Wq-specific repeat accumulation is paralleled by a dramatic increase in female-specific SNPs (Fig. 4C, D).

### Have structural rearrangements facilitated sex chromosome differentiation?

With respect to *G. affinis* sex chromosome morphology, both chromosome size and the positioning of the centromere indicate large-scale structural differentiation of the W-chromosome compared to Z. Because the W is by far the largest and also the only bi-armed, submetacentric element in *G. affinis*, and because its sister species *G. holbrooki* has only acrocentrics without differentiated sex chromosomes [21], we assume that the *G. affinis* W presents the evolutionarily derived, more differentiated chromosome type, compared to Z. We and others could explain the size difference between Z and W by the accumulation of evolutionary young repeat elements ([24], this study, Figs. 4 and 5), and of female-specific SNPs (Fig. 4). Strikingly, we could also demonstrate that these quantitative differences between Z and W are limited to the long arm of the W (Wq).

At first sight, the size difference between Z and W could prompt the assumption that Wq is simply a derived, female-specific addition to the ancestral acrocentric Z homologous Wp segment. However, when aligning W and Z sequences (Fig. 5; [24]) and also by oligopainting (Fig. 2), this hypothesis is refuted, because Z- and W-sequences are co-linear and share synteny over their entire length (Fig. 5). This is also supported by our CGH results, because GAF and GHO male-derived sequences were also found on Wq (Fig. 1). These findings predict large-scale structural rearrangements between inferred pre-Z and pre-W-chromosomes. Two alternative hypotheses can explain the gross structural differences, starting from an acrocentric autosome-like pair of undifferentiated pre-W and pre-Z: Either a centromeric shift by neocentromere formation or a pericentric inversion would be required to derive a structurally heterozygous pair of proto-Z and proto-W-chromosomes (Fig. 6).

Assuming end-to-end co-linearity and the absence of inversions as postulated by [24] (Fig. 6A), the hypothesis of evolutionary neocentromere formation on the W would be favored, through which the ancestral acrocentric pre-Z/W state would have evolved into the bi-armed proto-W (Fig. 6B). The ancestral pre-W terminal centromere at 0 Mbp of the reference sequence at an orthologous position to the acrocentric Z centromere would have been inactivated, concomitant with a presumably epigenetic neofunctionalization at an interstitial site at approx. 28 Mbp. These events would be accompanied by heterochromatinization and accumulation of centromeric repeats at the neocentromere (for review: [43]). Alternatively, considering an inverted orientation of Wq with respect to the orthologous Z-segment—a hypothetical scenario not supported by [24]—would require a whole arm pericentric inversion of the 0–28 Mbp Z-segment to align Z and W, and thereby moving the W centromere to its interstitial position (Fig. S3E).

Neocentromere formation in clinical cases and in evolution is well described (see [44] for review), including some examples from sex chromosomes in non-human mammals ([45] for review), but their role in sex chromosome evolution and differentiation is still unexplored. In contrast, inversions are prevalent in many taxa and were also implicated in sex chromosome evolution [46]. Both inversions and neocentromere formation may be drivers of sex chromosome differentiation suppressing recombination in the intervening segment (reviewed by [47], see also [21]), which in turn may promote mutation accumulation, sequence divergence and structural imbalances such as deletions or duplications. However, there are also some examples of nascent sex chromosomes, where recombination suppression seemingly predates structural rearrangements. Therefore, the lack of recombination may instead relax the selection against rearrangements ([48] and references therein).

## Conclusions

Vertebrate XY- and ZW-sex chromosome systems evolved numerous times in various clades and show extraordinary plasticity and evolutionary dynamics (reviewed by [1, 8, 9, 49]). The W in *G. affinis* is a prime example of an evolutionary young sex chromosome, which most parsimoniously emerged after the split from *G. holbrooki*, 2–7 Mya [26]. *G. holbrooki* would then have retained the ancestral pair of Z-like acrocentrics and an XY sex chromosome system with homomorphic gonosomes. Alternatively, assuming that the *G. affinis* WZ system is ancestral, the Wq expansion would have been reversed in *G. holbrooki*, combined with a WZ to XY turnover. Investigating the sex determination in the closely related *G. quadruncus and G. speciosa* may help distinguish between these alternatives [39].

In contrast to the Z and the short arm of the W (Wp), for the W long arm, we demonstrate the acquisition of sex-specific features, namely structural rearrangement, accumulation, and expansion of repetitive elements. Transcriptional activity throughout Wq further suggests the *G. affinis* W is a sex chromosome at its initial stage of differentiation with expansion and acquisition of novel genomic features, but not (yet) exhibiting degeneration and heterochromatization. This is in line with the classical evolutionary trajectory reviewed by [1].

Our cytogenomic data also point to transposable elements as a driver for the differentiation of Z and W in *G. affinis*, accompanied by large-scale structural rearrangement, and less by gene decay or sequence divergence. According to a standard model, structural differentiation and suppression of recombination between sex chromosomes would increase the mutational load of gonosomes and accelerate their genomic meltdown (e.g., [4] for review). However, an increasing body of evidence has recently relativized this classical paradigm (for review: [50]). In this work, we provided novel insight into the genomic organization and differentiation of evolutionary young sex chromosomes and thereby established a firm basis for further elucidating the functional significance of this intriguing evolutionary trajectory.

## Methods

### *Gambusia* tissue samples, cell culture, DNA and chromosome preparation

Tissue samples were obtained from *Gambusia affinis* (GAF, Rió Chuvíscar, Pena Blanca, Mexico) individuals. All tissue samples originated from the same batches of animals that were originally described by [26]. Three different primary epithelial cell cultures were established in parallel from a mixture of 14 unsexed embryos of developmental stages 24–25 according to the developmental stages of *Xiphophorus maculatus* as described in [51]. The cells were propagated in DMEM-medium supplemented with 15% fetal bovine serum, 10 mM sodium pyruvate, non-essential amino acids (Gibco BRL), 1000 U/ml penicillin–streptomycin, 5 mM 2-mercaptoethanol, and 2 mM glutamine at 28 °C in a humid atmosphere with 5% CO_2_ (following Kuhn et al., 1979). Chromosome preparations were obtained from two of these primary cultures at passages 13–15, following standard procedures. Both cultures showed a low polyploidy index of less than 10%, stable and inconspicuous chromosome counts, and a similar 70% female/30% male ratio as estimated from images captured in the various experiments with the W-chromosome present or absent.

Genomic DNA was prepared from two male and three female *G. affinis*, and from each two male and female *G. holbrooki* individuals, respectively. Sex-specific DNA mixes for *G. affinis* and *G. holbrooki* were prepared by pooling equal DNA amounts, designated GAFf, GAFm, GHOf, and GHOm, respectively. DNA from each of these four mixes was then subjected to whole-genome amplification (WGA) using the Illustra GenomiPhi V2 DNA Amplification Kit (GE Healthcare, Germany). These pooled and amplified genomic DNAs were used in all downstream applications, unless stated otherwise.

*G. affinis* testis and ovary cDNA libraries used in some PCR reactions and in comparative expressed sequence hybridization (CESH, see below) experiments were the same as described in [26].

### Classical staining techniques

Fluorescent R-banded *G. affinis* metaphases were obtained according to [52], using 7-amino-actinomycin D (7-AAD, Sigma, Germany). Metaphase slides were stained under a coverslip for 20 min at RT in 7-AAD at a final concentration of 20 ng/µl, followed by a brief rinse in 2 × SSC buffer at RT, and then embedding of the slide in Vectashield medium containing DAPI (Vector Laboratories, UK). C-banding was performed using the Ba(OH)_2_/Giemsa technique to stain constitutively heterochromatic chromosome regions, and Ag-NOR staining with AgNO_3_ and Giemsa to characterize active NORs (nucleolar organizing regions), following standard procedures for human chromosomes [53].

### DNA probe preparation for fluorescence in situ hybridization (FISH)

The *G. affinis* Z-chromosome oligopaint was obtained as a myTags™ 20 K labeled probe set from BioCat (Heidelberg, Germany) with technical support provided by Daicel Arbor Biosciences (Ann Arbor, MI, USA). This Z-chromosome oligopaint probe is made from a pool of approx. 20,000 evenly spaced 5′-ATTO-550-modified oligonucleotides that were designed from the repeat masked male *G. affinis* LG 01 sequence [24], resulting in an average density of one oligonucleotide per kb on the male LG 01 sequence. This probe was used in FISH according to the recommendations of the manufacturer, at a final concentration of 10 pmol/µl. Labeling of all other FISH probes with reporter molecules and the subsequent preparation for FISH were performed as described before [54]. WGA-amplified genomic DNA for the comparative genomic hybridization (CGH) experiments and WGA-amplified cDNA libraries for the CESH experiments were labeled by nick translation [54]. Genomic DNA and cDNA were labeled with Cy3-dUTP or biotin-dUTP, and co-hybridized in sets of two probes, as detailed in the results section. For each FISH experiment, all labeled probes were combined, ethanol co-precipitated with 20 µg of salmon sperm DNA, and then resuspended in hybridization buffer at a final concentration of 200 ng/µl hybridization mix per probe.

FISH probes for ten different microsatellite trinucleotide repeat motifs were prepared from 5′-fluorescence-modified DNA oligonucleotides, as described by [55]. We used probes (C)_30_ FITC, (A)_30_ Cy3, (GA)_15_ FITC, (CA)_15_ Cy3, (CAC)_10_ FITC, (TA)_15_ Cy3, (CGG)_10_ FITC, (GAG)_10_ Cy3, (GAC)_10_ FITC, and (CAT)_10_ Cy3 (Eurofins, Germany) in five different combinations of two probes each at a final concentration of 400 ng/µl hybridization mix per probe.

FISH probes, specific for 28S rDNA (NOR), were labeled by PCR-amplification from a mix of GAFf and GAFm template-DNA, using two different sets of primers and in the presence of biotin-dUTP or Cy3-dUTP. The first set of PCR primers was previously described by [32] (8f: GGGAAAGAAGACCCTGTTGAG, 11R: GCTTGGCBGCCACAAGCCAGTTA); the second set was designed from the *G. affinis* 28S sequence (GenBank AF152163.1; [56]: GAF28S-L1: CTGTAGTGGGCTCTCGGTTC, GAF28S-R1: AAGCCAGTTATCCCTGTGGT).

FISH probes for segments A/B/C/D/E/F/G/H/M/O/P/R of the *G. affinis amt* locus (Fig. 5) were also labeled by PCR in the presence of biotin-dUTP or Cy3-dUTP, using described primers [26]. In detail, the following *amt* gene segments were amplified: segment A (primer 9F/primer Gaf88R), B (7F/8R), C (4F/6R), D (Int3F/Int3R), E (1F/2R), F (8F/9R), G (6F/7R), H (Int3F/4R), M (Gaf88F/Gaf88R), O (8F/8R), P (7F/7R), and R (6R/6F).

Finally, FISH probes for transposable elements (TE) hAT-N145 and TC1-PP from *amt* intronic regions were designed from *G. affinis* aminomethyl transferase-like protein gene (partial coding sequence GenBank KP113677.1): hAT_F: AAAAGGTACAGTTCAGTAAAAC; hAT_R: ACTTGAGTGACTTTTGGATAA; TC1_F: GTCTTTTGGGACCAGAACACT; TC1_R: GGCGATCCACAGCTCAAGT. All PCR primers were synthesized by Eurofins MWG (Germany), PCR-cycles included initial denaturation at 95 °C for 3 min, 30 cycles (denaturation: 94 °C for 1 min; annealing 55 °C for 1.5 min; extension 72 °C for 2 min) and final extension at 72 °C for 10 min.

For each FISH experiment, all labeled PCR probes to be co-hybridized were combined (see the “Results” section), ethanol co-precipitated with 20 µg of salmon sperm DNA, and then resuspended in hybridization buffer at a final concentration of 500 ng/µl hybridization mix.

### Fluorescence in situ hybridization(FISH)

DNA probes resolved in hybridization buffer were denatured at 75 °C for 5 min. Next, the probe was added to the slide with the metaphase preparation, covered with a cover slip, and sealed with rubber cement. The slide was then denatured at 75 °C for 2 min in a Hybrite (VYSIS, USA) hybridization system, followed by hybridization at 37 °C overnight. Post-hybridization stringency washes included 2 × 5 min incubation in 0.1 × SSC buffer at 60 °C, except for microsatellite trinucleotide repeat and oligopaint probes. For the microsatellite probes, we used a 2-step protocol to evaluate hybridization efficiency, both at low and at high stringency. The first step included 2 × 5 min low stringency incubation in 2 × SSC buffer at RT according to the original protocol [55], followed by mounting in Vectashield embedding medium containing DAPI (Vector Laboratories, UK), and microscopic evaluation. Subsequently, Vectashield was removed, and the slides were washed again at high stringency for 2 min at 75 °C in 0.4 × SSC buffer. Post-hybridization stringency washes for the oligopaint probe included 1 × 5 min incubation in 2 × SSC buffer at 37 °C. Biotinylated probes were detected with Avidin-Alexa488 (Molecular Probes, USA), and finally, the slides were mounted in Vectashield embedding medium, containing DAPI (Vector Laboratories, UK).

### CGH and CESH

Both FISH techniques allow for a genome-wide, chromosome-centered comparison of DNA copy number differences between any two individuals using differentially labeled (e.g. red and green) sets of DNA probes [33, 35]. In CGH (comparative genomic hybridization) [35] deletions, amplifications and aneuploidies can be detected using genomic DNAs as probes, whereas CESH (comparative expressed sequence hybridization) [33] assays compare cDNA libraries and therefore expression differences. In CGH, two differentially labeled genomic DNA are co-hybridized [35], while cDNA libraries are being compared in CESH assays [33].

In CGH assays, color intensities along chromosomes correlate with probe sequence copy number in a sense that highly repetitive regions are more intensely stained than single-copy stretches of chromatin. Balanced regions show a label of the mixed color yellow, whereas regions of genomic imbalance in case of deletion or amplification in one of the two individuals, feature the color of the DNA showing a higher copy number. For example, when performing CGH using female DNA in green and male DNA in red in a euploid organism with a fully differentiated XY sex chromosome system, all autosomes would appear yellow with repeat-rich centromeres more intensely stained than single copy euchromatin, whereas the X would appear more green (2:1 XX:XY) and the Y red (0:1 XX:XY). Likewise, color intensities or color shifts observed in CESH assays are correlated with genome-wide expression levels and differential expression of genes positioned along chromosomes. In each CGH or CESH experiment, 200 ng of two differentially labeled genomic DNAs (CGH) or tissue-specific cDNA libraries (CESH), respectively, were mixed and hybridized in situ to *G. affinis* chromosomes as described above.

### Immuno-fluorescence detection of 5-methylcytosine

Hypermethylated chromosome segments were visualized by immuno-fluorescence (IF) staining of 5-methylcytosine (5mC). Metaphase slides were first treated with pepsin (Sigma, Germany) solution at a final concentration of 50 µg/ml 0.01N HCl for 3 min at 37 °C. We then performed a mock-FISH experiment as described above, followed by the application of a primary mouse-anti-5mC antibody (Diagenode, Belgium), according to the supplier’s instructions. After that, we applied the secondary antibody goat-anti-mouse-Atto550 (Sigma, Germany), followed by embedding in Vectashield antifade medium, containing DAPI (Vector Laboratories, UK).

### Sequence analysis of the *G. affinisamt* locus and primer design

To characterize the repeat content of the *amt* locus, we scanned the 6498 bp *amt* sequence of *G. affinis* (Genbank KP113677; [26]) for simple repeats, TEs, long non-coding RNAs, and micro-RNAs in public databases (girinst.org, noncode.org, fishtedb.org, rnacentral.org, repeatmasker.org and dfam.org; accessed April 2019, February 2023). We used the BLAST (https://blast.ncbi.nlm.nih.gov/Blast) and EMBOSS (www.ebi.ac.uk/Tools/emboss) web tools for *amt* local sequence alignments; PCR primers were designed using primer 3 (www.ncbi.nlm.nih.gov/tools/primer-blast). Exonerate (https://www.ebi.ac.uk/about/vertebrate-genomics/software/exonerate) was employed to align the protein sequence of *amt* from southern platyfish to the published female *Gambusia* assembly ([24]; chromosome W kept while Z removed). We set the parameter “–percent 10 –bestn 3” to Exonerate so fragments of the *amt* gene would also be detected.

### Bioinformatic analyses of the assembled W- and Z-chromosomes

DNA sequences of the W- and Z-chromosome were retrieved from a publicly available assembly of *G. affinis* [24]. These sequences were aligned using minimap2 (https://github.com/lh3/minimap) [40] with the parameter “asm5.” The sequence difference was then calculated with minimap2 and an in-house perl script (https://github.com/dukecomeback/maf2diff.pl) as the percentage of mutations in 10-kb sliding windows. SNPs and Indels were counted as one change.

To estimate and compare the extent of gene decay between the long (0–27.5 Mbp) and short arm (27.5–45 Mbp) of the W-chromosome, we collected 455,817 protein sequences from the vertebrate database of Swiss-Prot (https://www.uniprot.org/statistics/Swiss-Prot), RefSeq database (proteins with ID starting with “NP” from “vertebrate_other”), and the NCBI genome annotation of human (GCF_000001405.39_GRCh38), zebrafish (GCF_000002035.6), platyfish (GCF_002775205.1), medaka (GCF_002234675.1), mummichog (GCF_011125445.2), turquoise killifish (GCF_001465895.1), and guppy (GCF_000633615.1) and mapped them on the W-chromosome using GeneWise (https://www.ebi.ac.uk/Tools/psa/genewise/). GeneWise was used with default parameters and “-pseudo -genesf” which only affects the layout of the output. For saving the running time of GeneWise, we located the rough alignment area ahead GeneWise using genblastA with the parameter “-e 1e-5 -c 0.5 -r 1.” For further analysis, we only kept the alignment results with alignment coverage > 35% and a percentage of identity > 50%.

The repeats on chromosomes W and Z and their Kimura value (determined with divCpGMod) were annotated using RepeatMasker (www.repeatmasker.org). We selected repeats with Kimura < 1 on W and Z and plotted them in a bubble chart with the size referring to the repeat length.

Pool sequencing data from a previous study were downloaded from [22] and aligned to the genome using the bioinformatic pipeline described therein. To confirm the Wq-specific FISH signal with the hAT-related probe, we took the respective primer sequences, aligned, and located them on the assembled sequences of both W and Z [24].

### Microscopy and image analysis

The microscopic evaluation of C-banding, Ag-NOR staining and of FISH experiments was carried out using an Axioplan 2 Imaging microscope (Zeiss, Germany), equipped with a brightfield/phase contrast unit and an epifluorescence setup, including fluorescence filter sets for DAPI, DEAC, FITC, Cy3, TexasRed, and Cy5. We analyzed at least ten male and female metaphases per experiment. Quantitative image densitometric analysis and chromosome arm length ratio measurements were performed using the Image J software (NIH, US) version 1.48v color profiler and line analyzer tool, respectively, analyzing at least ten metaphases per experiment.

## Supplementary Information

**Additional file 1: Fig. S1.** CoAQmparative Genomic Hybridization using genomic DNA from *Gambusia affinis* and *G. holbrooki* male and female individuals. A) and B) Examples of *G. affinis* female and male metaphase spreads after CGH using differentially labeled combinations of genomic DNA as indicated. Arrowheads highlight the W-chromosome in female metaphase spreads, asterisks mark a medium sized acrocentric pair in males and females showing a long arm subtelomeric repeat cluster overrepresented in *G. holbrooki*.

**Additional file 2: Fig. S2.** Genomic features of the *G. affinis* W-chromosome. A) - C) FISH using fluorescent oligonucleotide repeat probes. A) C_30_ green/A_30_ red, B) GA_15_ green/CA15_15_ red and C) CGG_10_ green/GAG_10_ red. No microsatellite repeat clusters were detected at the resolution of FISH, and in particular not on the W-chromosome. Only pericentromeric regions of some chromosomes showed enrichment for the poly-A motive as indicated by asterisks in A). In C) distal Wq harbors a stretch of CGG-repeats. D) Combined staining of GC-rich regions using 7-AAD and AT-rich regions using DAPI showing a trend towards more CG-rich centromeres. E) C-banding was restricted to centromeric regions of all chromosomes, Wq is C-band negative and therefore not heterochromatic. F) and G) partial *G. affinis* metaphases after Ag-NOR staining. Active NORs marked by asterisks were found in the subtelomeric region of Wq, and on up to six acrocentric chromosomes. H) and I) these findings were confirmed by FISH using 28S rDNA PCR products. In addition to rDNA positive regions on Wq and on pericentromeric regions of some acrocentric chromosomes, cross-hybridization to all centromeric regions indicates genomic proximity of rDNA and centromeric sequences.

**Additional file 3: Fig. S3.** Sequential DAPI/7-AAD staining and FISH using a Z-specific oligopaint probe and *G. affinis* gDNA facilitated sex chromosome identification. A) ZW female and B) ZZ male metaphase with false colored overlay of GC-specific 7-AAD stain in red, AT-specific DAPI stain in green, the Z-oligopaint in magenta and *G. affinis* gDNA in cyan. C) Karyogram of the DAPI/7-AAD stained chromosomes from B), and D) comparison of sex chromosomes from A) and B), scaled to size, demonstrating that the Z-chromosome is a large acrocentric, and the W-chromosome is significantly larger than the Z. Please note that the W is upside down so homologous regions are aligned. E) two alternative hypotheses on evolutionary structural chromosome rearrangements starting from a pre-W resembling an acrocentric autosome similar in structure and homologous to an inferred pre-Z: in a first step, the pre-W becomes structurally rearranged by a centromeric shift concomitant with neocentromere formation or by a pericentric inversion, resulting in a proto-W chromosome. Available genome sequencing data [24] favor hypothesis 1, although at present hypothesis 2 cannot be refuted entirely. For an illustration of subsequent steps of W chromosome differentiation, please refer to Figure 6B.

**Additional file 4: Fig. S4.** A) and B) FISH mapping of fluorescent PCR products from exonic and intronic regions of the *amt* locus to female and male* G. affinis* metaphases. See Figure 5E for FISH probe description.

**Additional file 5: Table S1.** Gene duplications on the W chromosome compared to Z. **Table S2.** micro-RNA, long noncoding RNA and repeat content of the *amt* locus.

## Data Availability

All data generated or analyzed during this study are included in this published article.

## Abbreviations

5mC: 5-Methylcytosine
7-AAD: 7-Amino-actinomycin D
*amt*: Aminomethyl transferase
cen: Centromere
CESH: Comparative expressed sequence hybridization
CGH: Comparative genomic hybridization
DAPI: 4′,6-Diamidino-2-phenylindol
FI: Fluorescence intensity
FISH: Fluorescence in situ hybridization
GAF: *Gambusia affinis*
GHO: *Gambusia holbrooki*
IF: Immuno-fluorescence
LG: Linkage group
NOR: Nucleolar organizer region
p-arm: Short (chromosome) arm
PCG: Protein coding gene
q-arm: Long (chromosome) arm
rDNA: Ribosomal DNA
SNP: Single nucleotide polymorphism
SSC: Saline sodium citrate
TE: Transposable element
WGA: Whole-genome amplification
