# Supplementary figures and images for: Massive expansion of sex-specific SNPs, transposon-related elements, and neocentromere formation shape the young W-chromosome from the mosquitofish Gambusia affinis
Source: BMC Biol. 2023 May 15;21:109. doi: 10.1186/s12915-023-01607-0 (PMC10186657; doi:10.1186/s12915-023-01607-0)

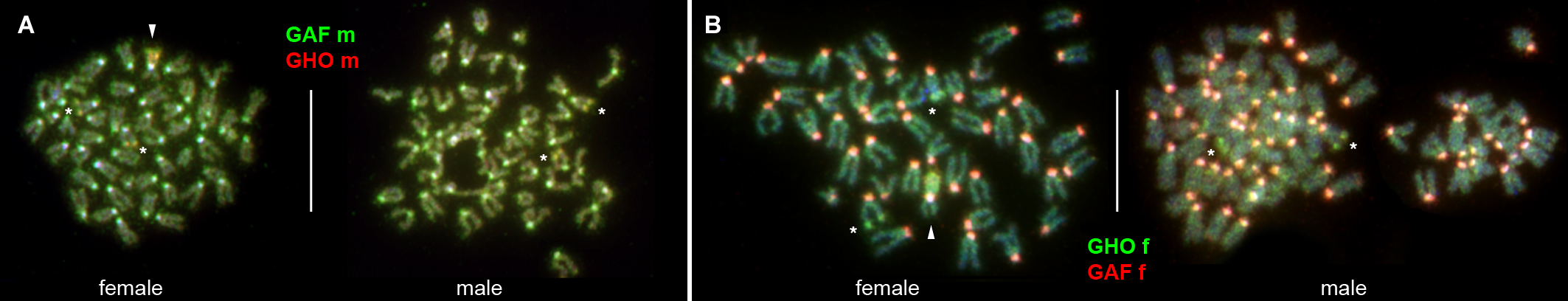

Supplement: Supplementary file 1 — Additional file 1: Fig. S1. CoAQmparative Genomic Hybridization using genomic DNA from Gambusia affinis and G. holbrooki male and female individuals. A) and B) Examples of G. affinis female and male metaphase spreads after CGH using differentially labeled combinations of genomic DNA as indicated. Arrowheads highlight the W-chromosome in female metaphase spreads, asterisks mark a medium sized acrocentric pair in males and females showing a long arm subtelomeric repeat cluster overrepresented in G. holbrooki. [file 12915_2023_1607_MOESM1_ESM.tif]

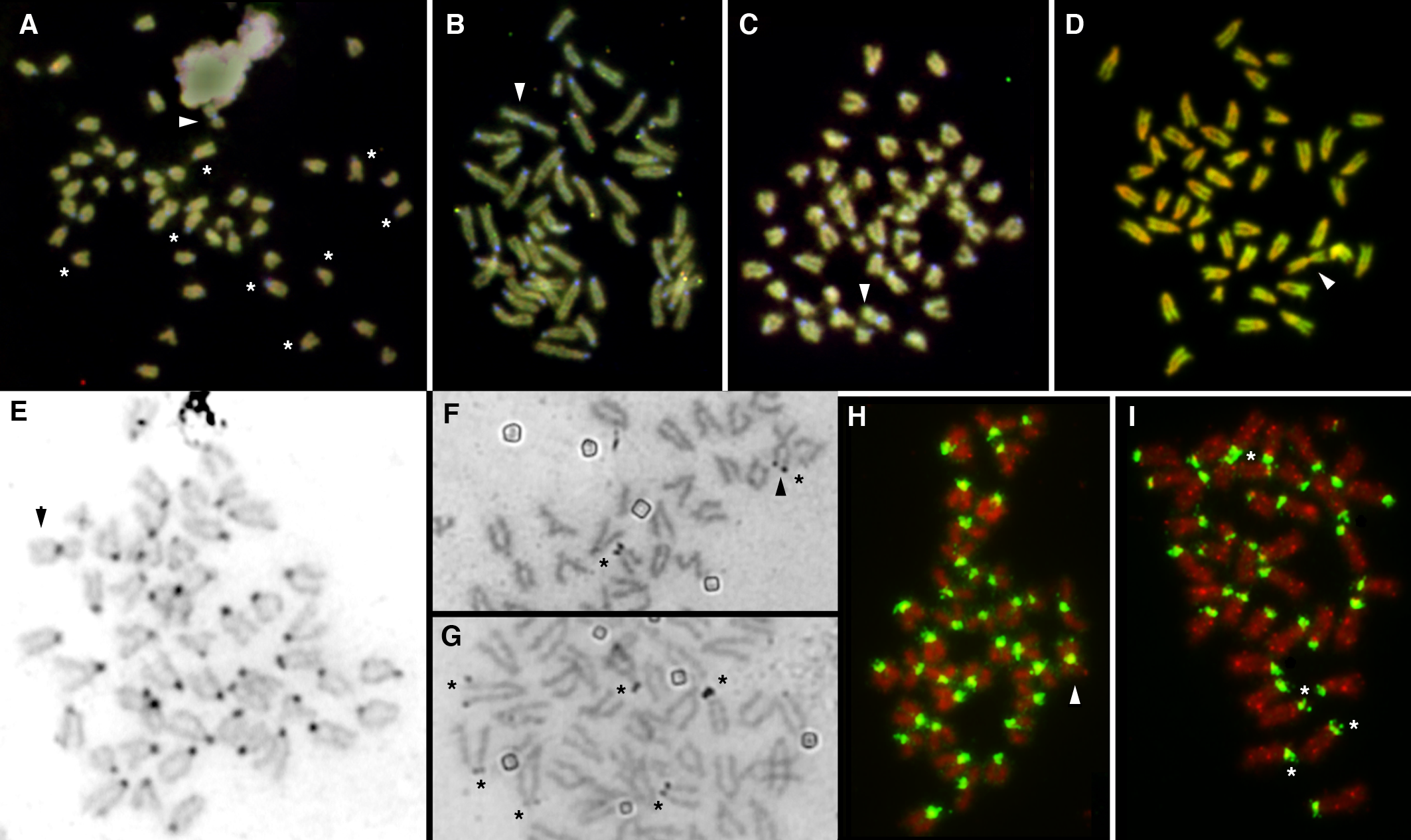

Supplement: Supplementary file 2 — Additional file 2: Fig. S2. Genomic features of the G. affinis W-chromosome. A) - C) FISH using fluorescent oligonucleotide repeat probes. A) C30 green/A30 red, B) GA15 green/CA1515 red and C) CGG10 green/GAG10 red. No microsatellite repeat clusters were detected at the resolution of FISH, and in particular not on the W-chromosome. Only pericentromeric regions of some chromosomes showed enrichment for the poly-A motive as indicated by asterisks in A). In C) distal Wq harbors a stretch of CGG-repeats. D) Combined staining of GC-rich regions using 7-AAD and AT-rich regions using DAPI showing a trend towards more CG-rich centromeres. E) C-banding was restricted to centromeric regions of all chromosomes, Wq is C-band negative and therefore not heterochromatic. F) and G) partial G. affinis metaphases after Ag-NOR staining. Active NORs marked by asterisks were found in the subtelomeric region of Wq, and on up to six acrocentric chromosomes. H) and I) these findings were confirmed by FISH using 28S rDNA PCR products. In addition to rDNA positive regions on Wq and on pericentromeric regions of some acrocentric chromosomes, cross-hybridization to all centromeric regions indicates genomic proximity of rDNA and centromeric sequences. [file 12915_2023_1607_MOESM2_ESM.tif]

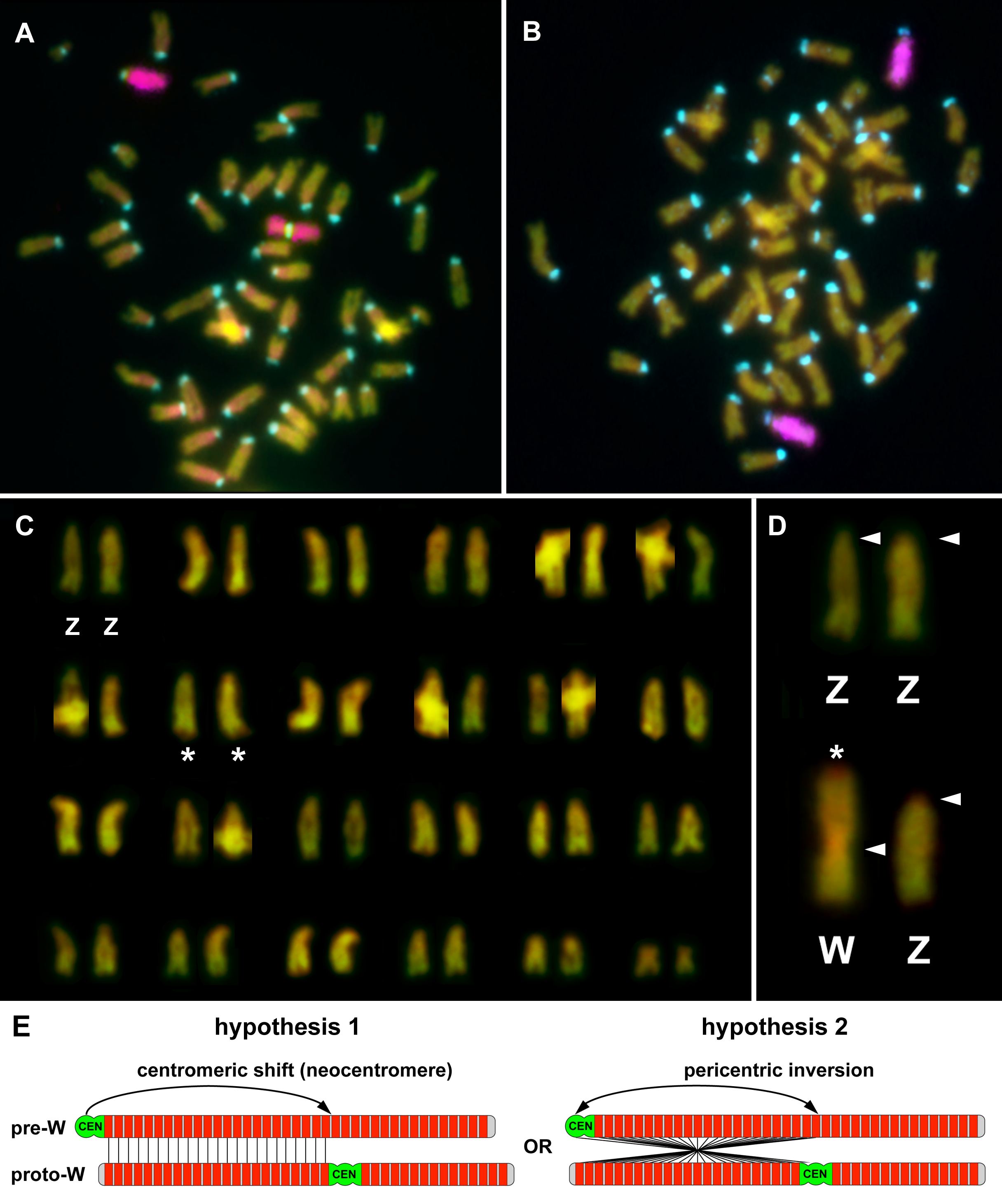

Supplement: Supplementary file 3 — Additional file 3: Fig. S3. Sequential DAPI/7-AAD staining and FISH using a Z-specific oligopaint probe and G. affinis gDNA facilitated sex chromosome identification. A) ZW female and B) ZZ male metaphase with false colored overlay of GC-specific 7-AAD stain in red, AT-specific DAPI stain in green, the Z-oligopaint in magenta and G. affinis gDNA in cyan. C) Karyogram of the DAPI/7-AAD stained chromosomes from B), and D) comparison of sex chromosomes from A) and B), scaled to size, demonstrating that the Z-chromosome is a large acrocentric, and the W-chromosome is significantly larger than the Z. Please note that the W is upside down so homologous regions are aligned. E) two alternative hypotheses on evolutionary structural chromosome rearrangements starting from a pre-W resembling an acrocentric autosome similar in structure and homologous to an inferred pre-Z: in a first step, the pre-W becomes structurally rearranged by a centromeric shift concomitant with neocentromere formation or by a pericentric inversion, resulting in a proto-W chromosome. Available genome sequencing data [24] favor hypothesis 1, although at present hypothesis 2 cannot be refuted entirely. For an illustration of subsequent steps of W chromosome differentiation, please refer to Figure 6B. [file 12915_2023_1607_MOESM3_ESM.tif]

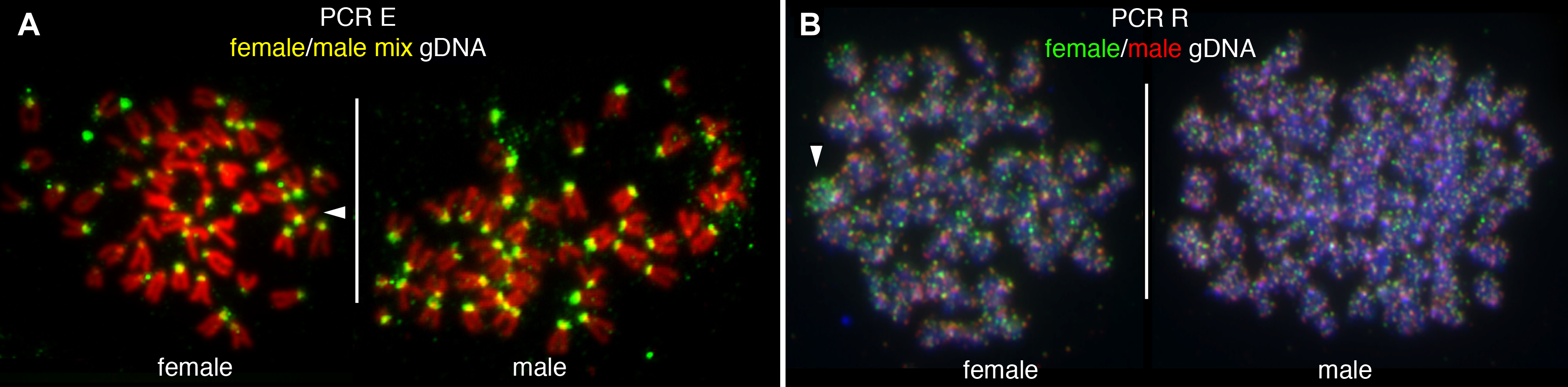

Supplement: Supplementary file 4 — Additional file 4: Fig. S4. A) and B) FISH mapping of fluorescent PCR products from exonic and intronic regions of the amt locus to female and male G. affinis metaphases. See Figure 5E for FISH probe description. [file 12915_2023_1607_MOESM4_ESM.tif]
