# Supplementary material for: Massive expansion of sex-specific SNPs, transposon-related elements, and neocentromere formation shape the young W-chromosome from the mosquitofish Gambusia affinis
Source: BMC Biol. 2023 May 15;21:109. doi: 10.1186/s12915-023-01607-0 (PMC10186657; doi:10.1186/s12915-023-01607-0)
Supplement: Supplementary file 5 — Additional file 5: Table S1. Gene duplications on the W chromosome compared to Z. Table S2. micro-RNA, long noncoding RNA and repeat content of the amt locus. [file 12915_2023_1607_MOESM5_ESM.pdf]

Supplementary Table 1: Gene duplications on the W chromosome compared to Z

gene identification on Z			copy number on W	Aliases description
query	alignment coverage	percentage of identity		
XP_005798978.1	100	99,33	3	tubulin alpha-1B chain
XP_023187410.1	100	95,73	2	RNA binding motif protein 10
XP_023186262.1	100	96,58	2	procollagen-lysine, 2-oxoglutarate 5-dioxygenase 1a
XP_005801475.1	100	90,11	2	RIB43A domain with coiled-coils 1
XP_023197040.1	100	97,08	2	polyhomeotic homolog 2a (Drosophila)
XP_023197622.1	100	92,49	2	forkhead box protein P3-like
XP_005808727.1	100	96,34	2	potassium voltage-gated channel subfamily A member 10-like
XP_008411034.1	100	96,46	2	solute carrier family 35 member C2
XP_008411025.1	100	99,2	2	TATA-box binding protein associated factor 13
XP_005808712.1	96,89	90,06	2	leucine rich repeat containing 23
XP_005800223.1	100	95,87	2	choline dehydrogenase
XP_005808725.1	100	95,31	2	ETS variant transcription factor 7
XP_017161451.1	92,57	99,07	2	nuclear transcription factor Y, alpha, like
XP_008411189.1	100	97,23	2	mitofusin 2
XP_023197455.1	100	97,86	2	zinc finger protein PLAG1-like
XP_005808731.1	100	96,53	2	ubiquitin specific peptidase 21
XP_005815856.1	100	94,48	3	LAS1 like ribosome biogenesis factor
XP_014327841.1	100	86,67	2	patatin-like phospholipase domain-containing protein 2
XP_014326788.1	95,93	95,41	2	myelin transcription factor 1-like
XP_023191973.1	96,39	97,29	2	TBC1 domain family, member 22B
XP_005804892.3	100	95,44	2	thiamin pyrophosphokinase 2

Supplementary Table 2: micro-RNA, long noncoding RNA and repeat content of the *amt* locus (http://www.mirbase.org, https://www.girinst.org https://rnacentral.org, http://www.noncode.org/, http://www.fishtedb.org)

name	type	*amt* region	*amt* position nt	source	FISH (probe)
Exon 1			1-74	Lamatsch et al. 2015	- (E)
Harbinger-N174_Croc	TE	exon 1	24-67	repbase	- (E)
miR-301	Micro-RNA	Intron 1	134-155	mirbase	- (E)
$(T)_3GTC(T)_7AA(T)_3A(T)_6W(T)_6AG(T)_4C$	Simple repeat	Intron 1	251-289	repeatmasker	- (E)

Exon 2			1517-1324	Lamatsch et al. 2015	- (E)
Exon 3			1451-1531	Lamatsch et al. 2015	n.d.
hAT-N145_DR	TE	Intron 3	1896- 1985	repbase	+ (D/hAT)
Amazon_molly_rnd-4_family-1248#DNA/hAT-Charlie	TE	Intron 3	2040-2395	FishTEDB	+ (D)
miR-19	Micro-RNA	Intron 3	2159-2185	mirbase	+ (D)
Gypsy-9_OD-I	TE	Intron 3	2266-2327	repbase	+ (D)
H. sapiens NONHSAT070712	lncRNA	Intron 3-Exon 4	2607-2919	RNAcentral	+ (D/H)
Exon 4			2684-2849	Lamatsch et al. 2015	+ (H)
$(TG)_{11}YGYG(TG)_3YG(TG)_5$	Simple repeat	Intron 4	2873-2917	repeatmasker	- (C)
H. sapiens lnc-C15orf59-1:3<br>*H. sapiens* lnc-ZNF705D-2:19<br>*H. sapiens* lnc-C6orf112-6:1<br>*M. musculus* OTTMUST00000023722.1	lncRNA	Intron 4-Intron 5	2920-3046	RNAcentral	- (C)
Exon 5			2930-3007	Lamatsch et al. 2015	- (C)
Exon 6			3168-3316	Lamatsch et al. 2015	+ (R/G)
Gypsy-14_GA-I	TE	Exon 6	3177- 3236	repbase	+ (R/G)
TC1_PP	TE	Intron 6	3783-3994	repbase	- (TC1)
$(TCTG)_5$	Simple repeat	Intron 6	4073-4093	repeatmasker	+ (G)
Helitron-like-4a_Hmel:	TE	Intron 6	4065- 4097	repbase	+ (G)
Exon 7			4103-4283	Lamatsch et al. 2015	+ (P/B)
H. sapiens NONHSAT097211	lncRNA	Exon 7	4148-4285	RNAcentral	+ (P/B)

Exon 8			4444-4599	Lamatsch et al. 2015	- (F/O)
H. sapiens NONHSAT097211	lncRNA	Exon 9	5089-5262	RNAcentral	- (F/A)
Exon 9			5094-5270	Lamatsch et al. 2015	- (F/A)
miR-103	Micro-RNA	Intron 9 UTR	5445-5460	mirbase	- (A)
SINE2-1_DL	TE	Intron 9 UTR	5627- 5774	repbase	- (A)
DNA-8-3_HM	TE	Intron 9 UTR	6203- 6255	repbase	+ (M)
